# Gut microbiota non-convergence and adaptations in sympatric Tibetan and Przewalski’s gazelles

**DOI:** 10.1016/j.isci.2024.109117

**Published:** 2024-02-06

**Authors:** Pengfei Song, Feng Jiang, Daoxin Liu, Zhenyuan Cai, Hongmei Gao, Haifeng Gu, Jingjie Zhang, Bin Li, Bo Xu, Tongzuo Zhang

**Affiliations:** 1 Key Laboratory of Adaptation and Evolution of Plateau Biota, Northwest Institute of Plateau Biology, Chinese Academy of Sciences, Xining, Qinghai 810008, China; 2 University of Chinese Academy of Sciences, Beijing 100049, China; 3 Qinghai Provincial Key Laboratory of Animal Ecological Genomics, Xining, Qinghai 810008, China; 4 Qinghai University, Xining, Qinghai 810016, China

**Keywords:** Wildlife microbiology, Zoology, Microbiology, Microbiome

## Abstract

Unraveling the connection between gut microbiota and adaptability in wild species in natural habitats is imperative yet challenging. We studied the gut microbiota of sympatric and allopatric populations of two closely related species, the *Procapra picticaudata* and *P. przewalskii*, with the latter showing lower adaptability and adaptive potential than the former. Despite shared habitat, sympatric populations showed no convergence in gut microbiota, revealing distinct microbiota-environment relationships between the two gazelle species. Furthermore, the gut microbiota assembly process of the *P. przewalskii* was shifted toward homogeneous selection processes relative to that of the *P*. *picticaudata*. Those taxa which contributed to the shift were mainly from the phyla *Firmicutes* and *Verrucomicrobiota*, with functions highly related to micronutrient and macronutrient metabolism. Our study provides new insights into the complex dynamics between gut microbiota, host adaptability, and environment in wildlife adaptation and highlights the need to consider host adaptability when examining wildlife host-microbiome interplay.

## Highlights

• Sympatric Tibetan and Przewalski’s gazelles showed non-convergence of gut microbiota
• Gut microbiota-environment interactions are influenced by host adaptability
• Przewalski’s gazelle need more specialized gut microbiota than Tibetan gazelle to adapt

## Graphical abstract

**Figure:**
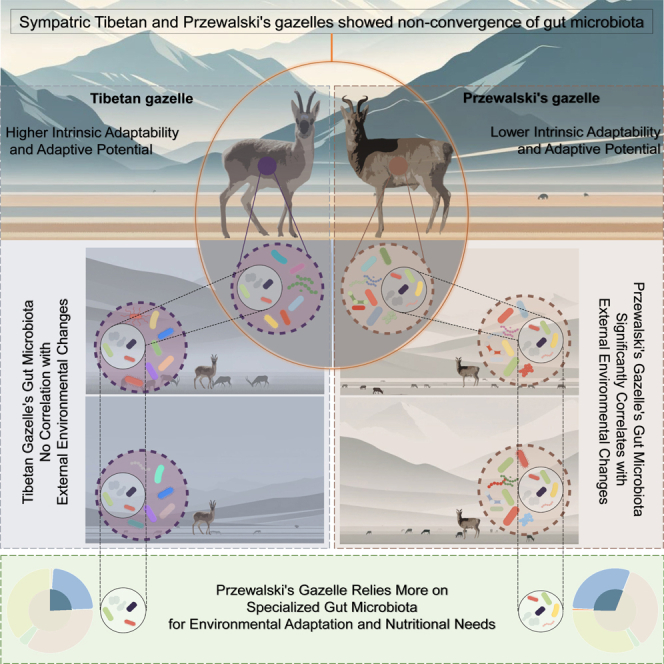

## Introduction

The adaptability of species to their environment is crucial for conservation biology, as it guides management decisions and strategies to ensure the long-term survival of endangered species facing environmental challenges. For a healthy individual of a wild species living in its natural distribution area, it can be considered adapted to its current environment, as implied by the host-gut microbiota co-speciation,^1^ where the gut microbiota and its host have achieved homeostasis. Gut microbiota can significantly impact host fitness.^2^^,^^3^ However, a seemingly obvious but fundamentally unresolved question remains. To what extent does the adaptation of this species to its environment rely on the services provided by its gut microbial community? Addressing this question is fraught with challenges. Conducting research on wild animals, especially endangered species, is inherently difficult due to ethical considerations and the logistical constraints of performing controlled experiments in the field.^4^ Additionally, as host-associated gut microbiota in wildlife is typically influenced by a complex interplay of several key factors, including phylogenetic relatedness between hosts,^5^ environmental variation,^6^ and host characteristics.^7^^,^^8^

In terms of phylogeny, studies have demonstrated that in hosts with higher phylogenetic relationships (e.g., class, order, or family level), the gut microbial composition is highly correlated with the host phylogeny.^9^^,^^10^ However, with closer phylogenetic relationships between hosts (e.g., genus, species level), it has become insufficient to predict gut microbial composition. At this point, gut microbiota composition is more substantially influenced by the host’s environment and other factors.^6^^,^^11^^,^^12^ Environmental heterogeneity increased with geographic distance and, in the vast majority of studies, could explain or even predict changes in gut microbial composition and diversity, generally attributed to differences in physical factors or biological factors of the population living in diverse environments.^6^^,^^11^ Thus, an emerging consensus is that changes in gut microbial composition and diversity of closely related species are most likely dominated by environmental changes, which also implies that a host’s external ecology shapes its internal ecology with consequences for the “ecosystem services” microbiomes provide to hosts.^6^ A reasonable inference, therefore, is that if hosts are closely related phylogenetically, strictly co-distributed in the same geographic area, and possess highly similar ecological habits, then it can be predicted that their gut microbial composition and diversity should be highly similar.

Comparative studies of sympatric and allopatric populations between closely related species provide a framework that differentiates the effects of environmental factors on gut microbiota from those of host heritable factors, and vice versa.^13^ Fortunately, among the numerous wild species inhabiting the Qinghai-Tibet Plateau, the congeneric Tibetan gazelle (*Procapra picticaudata*) and the Przewalski’s gazelle (*P. przewalskii*), with highly similar genetic backgrounds, are well-suited for such a study. The Przewalski’s gazelle is an endangered species with a restricted distribution in Qinghai Province, China,^14^ while the Tibetan gazelle is widely distributed across the Qinghai-Tibet Plateau.^15^ The potential suitable habitat area for Przewalski’s gazelle is only 9.7% of that for Tibetan gazelle,^16^ reflecting a significantly lower intrinsic adaptability of Przewalski’s gazelle than Tibetan gazelle. Moreover, the fact that Przewalski’s gazelle has historically experienced a population bottleneck, leading to a significant decline in its population and a marked reduction in genetic diversity compared to Tibetan gazelle,^17^^,^^18^ suggests that the adaptive potential of Przewalski’s gazelle is lower than that of Tibetan gazelle. In the Shengge Area upon Upper Buha River, located in the northwest region of the Qinghai Lake basin, both species exhibit a strictly sympatric distribution,^19^ with highly similar dietary preferences,^20^ habitat utilization, social structure, and activity patterns, approximately 70% of their total home range is overlapping.^21^

The relative proportion of deterministic and stochastic processes in a community can provide insights into the underlying mechanisms that shape community structure and function.^22^^,^^23^ In general, deterministic processes can refer to the host filtering the microbial community based on factors such as diet and physiology, as well as biotic interactions such as competition, mutualism, and predation among the microbial species in the gut ecosystem.^24^ These niche-based mechanisms shape the composition and structure of the gut microbiota community, creating a specific environment that favors certain bacterial species over others based on their functional traits and ecological niches.^25^ In contrast, stochastic processes such as the random colonization of new bacterial species, or the random fluctuations in birth and death rates of different microbial species, play an important role in gut microbiota assembly. These neutral-based mechanisms can lead to unpredictable changes in the composition and abundance of gut microbiota over time, even in the absence of selective pressures from the host environment.^24^ A high proportion of deterministic processes suggest that ecological selection (by biotic and/or abiotic factors) is driving community assembly.^26^ The assembly of gut microbiota, particularly in endangered species, is a relatively understudied area of research in microbial ecology. We believe that it is partly due to the concepts of deterministic and stochastic processes have not been well integrated with host-microbe interactions.

We hypothesize that if no significant convergence in gut microbial diversity and similarity is observed between sympatric Tibetan gazelle and Przewalski’s gazelle compared to their allopatric counterparts, then the differences in the intrinsic adaptability and adaptive potential between the closely related species could counteract the homogenizing effect of a shared environment. Furthermore, we also predict that the correlation between gut microbiota and the environment can vary significantly due to differences in host intrinsic adaptability and adaptive potential for Tibetan gazelle and Przewalski’s gazelle, leading to distinct responses to environmental changes and diverse ecological services provided by their gut microbiota.

To test the aforementioned hypotheses, fecal samples of sympatric Tibetan gazelle and Przewalski’s gazelle were collected from the Shengge Area upon Upper Buha River, and fecal samples of allopatric Tibetan gazelle and Przewalski’s gazelle populations at similar geographic distances from where they were living sympatrically, respectively. Extrinsic microbes are known to influence gut microbial composition and diversity.^27^ According to our field observations, both gazelle species digging and ingesting plant roots suggest direct ingestion of soil microbes. Additionally, food may also serve as vectors for environmental microbes to enter the animal gut.^12^ Therefore, we also collected soil samples from the corresponding locations to characterize the environmental microbiota in order to investigate the potential impact of environmental microbial communities on gut microbiota diversity. To comprehensively characterize the gut microbiota of both Przewalski’s gazelle and Tibetan gazelle, we conducted studies on all bacterial taxa, as well as on the abundant and rare taxa. Previous studies have shown that these different groups of taxa have different distribution patterns and may respond differently to environmental changes.^28^^,^^29^

## Results

**Figure 1:**
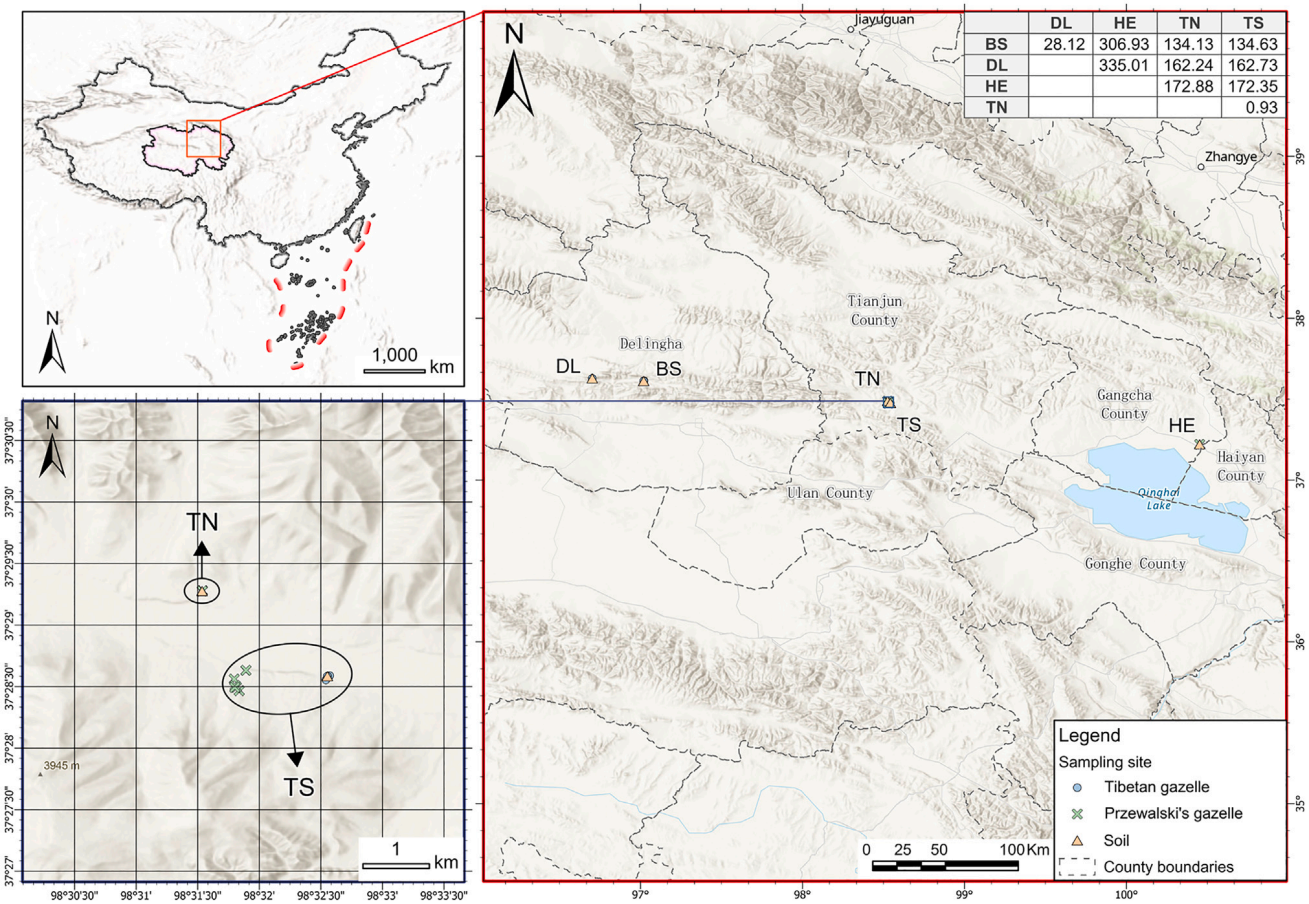
Location of sampling sites for gazelles and soil samples in Qinghai Province, China Map showing the 6 sampling locations (BS, DL, TN, TS, HE) of gazelles and soil samples, the solid blue circle denoted fecal samples of Tibetan gazelle, the solid green cross denoted fecal samples of Przewalski’s gazelle, and the solid orange triangle denoted soil samples. The top right-hand corner is a matrix of distances (kilometers) between each sampling site. The bottom left is one of the study areas, Shengge Area upon Upper Buha River, Tianjun County, Qinghai Province, China, where Tibetan gazelle and Przewalski’s gazelle coexist. See also Table S1 and S2.

A map of all sampling locations is presented in Figure 1, and detailed sample information is presented in Table S1. The sampling area and sites were geocoded with ArcGIS pro (V3.0.1). Altogether, the sample set (Table S2) allowed comparisons of 1 pair of sympatric congeneric populations (prTS Vs. prTN), 2 pairs of sympatric heterogenetic populations (piTS Vs. prTS and piTS Vs. prTN), 5 pairs of geographically separated congeneric populations (piBS Vs. piDL, piBS Vs. piTS, piDL Vs. piTS, prHE Vs. prTN, and prHE Vs. prTS), and 7 pairs of geographically separated heterogenetic populations (piBS Vs. prHE, piBS Vs. prTN, piBS Vs. prTS, piDL Vs. prHE, piDL Vs. prTN, piDL Vs. prTS, and piTS Vs. prHE).

### Overall trends in bacterial composition

A total of 16,483,828 (228,942 reads/sample) raw reads were generated for 57 fecal and 15 soil samples, respectively. After quality filtering, a total of 4,124,307 16S rRNA V3–V4 gene sequences, ranging from 26,685 (sTN_01) to 88,818 (prTN_08) in each sample, and 6,945 amplicon sequence variants (ASVs) were detected across all samples. To reduce the heterogeneity, the filtered ASV table was rarefied to the minimum sequencing depth of all samples (depth = 26,685) (see Figure S1 for details). As the sequencing depth increased, the rarefaction curves of the observed ASVs (*S*_*ob*s_) and the Shannon-Wiener index (Shannon) of ASVs gradually reached a plateau (Figure S2), which suggests that the sequencing depth was sufficient to reflect the bacterial diversity of each sample.

**Figure 2:**
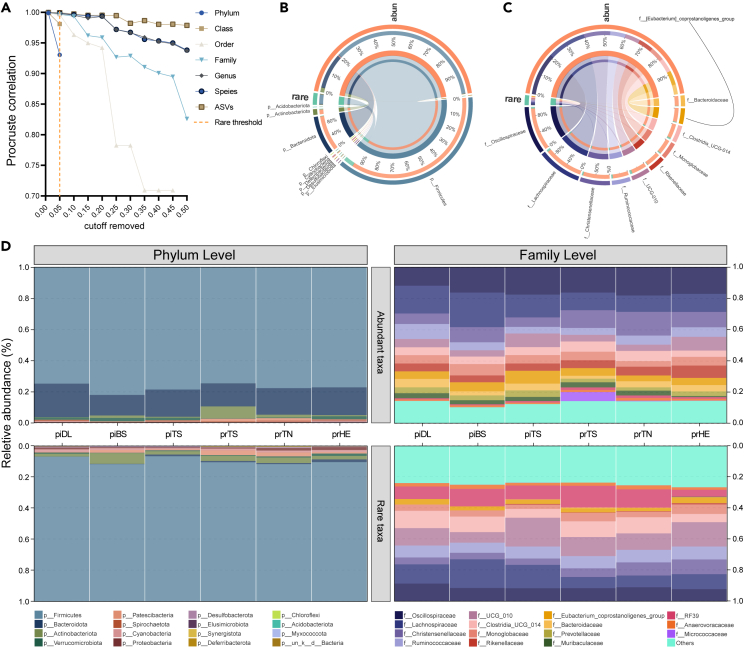
General patterns of the bacterial profile (A) Identification of rare threshold using multivariate cut-off level analysis (MultiCoLA) across Tibetan gazelle and Przewalski’s gazelle. (B and C) General description of abundant and rare ASVs datasets at phylum and family level, respectively. (D) Relative abundance of microbial phylum and family level averaged per population. Populations are listed from west to east. See also Tables S3 and S4 and Figure S4.

The rare taxa threshold in gazelles was defined as 5% of the total number of sequences (Figures 2A and S3). In total, 2,835 (53.0%) ASVs with 1,445,034 sequences were considered abundant ASVs, mainly coming from *Firmicutes* (2,429 ASVs), *Bacteroidota* (263 ASVs), *Actinobacteriota* (31 ASVs), and *Patescibacteria* (31 ASVs). While 2,512 (47.0%) ASVs with 76,011 sequences were classified as rare ASVs, mainly from *Firmicutes* (2,009 ASVs), *Actinobacteriota* (220 ASVs), *Proteobacteria* (114 ASVs), *Cyanobacteria* (33 ASVs), *Bacteroidota* (32 ASVs), and *Patescibacteria* (30 ASVs) (Figures 2B and 2C).

### Gut microbiota differs between host and populations

#### Community structure

A total of 4,282 and 5,056 ASVs were identified in the gut microbiota of Tibetan gazelle and Przewalski’s gazelle, respectively. In addition, we investigated the core ASVs in both host species, defined as those present in at least 90% of samples. Results revealed that these core ASVs all belonged to abundant taxa. Specifically, 275 core ASVs comprising 245,588 reads were found in Tibetan gazelle, primarily assigned to *Firmicutes* (266 ASVs) and *Bacteroidota* (5 ASVs). For Przewalski’s gazelle, 371 core ASVs comprising 317,747 reads were identified, belonging mainly to *Firmicutes* (342 ASVs), *Bacteroidota* (18 ASVs), *Actinobacteriota* (3 ASVs), and *Patescibacteria* (3 ASVs). Among the core ASVs, 157 ASVs were shared between the two gazelle species, which were primarily from *Firmicutes* (153 ASVs) and *Bacteroidota* (2 ASVs). The taxonomy of core ASVs is detailed in Table S3.

The microbial composition of abundant and rare taxa at the phylum and family levels in Tibetan gazelle and Przewalski’s gazelle are detailed in Table S4. For abundant phyla (Figure 2D), *Firmicutes* (74.7%–82.1%; min–max) and *Bacteroidota* (13.2%–21.5%) were predominant (relative abundance > 1%) in both gazelles. For rare phyla, the predominant taxa were *Firmicutes* (87.2%–93.1%), *Actinobacteriota* (2.0%–6.5%) and *Cyanobacteria* (1.0%–1.6%). At family level (Figure 2D), for abundant taxa, 11 families including *Oscillospiraceae* (11.8%–18.1%), *Lachnospiraceae* (10.5%–22.2%), *Christensenellaceae* (6.1%–15.2%), and *Ruminococcaceae* (4.2%–9.6%) were predominant across all populations. For rare taxa at family level, the predominant families were *Oscillospiraceae* (7.7%–11.2%), *Lachnospiraceae* (7.2%–17.8%), *Oscillospirales UCG-010* (7.1%–17.1%), *Ruminococcaceae* (6.7%–8.3%), etc.

In addition, each population’s shared and unique bacterial taxa were also assessed. Among 11 abundant and 18 rare bacterial phyla, 10 and 9 were shared by all populations, respectively (Figure S4), and *Planctomycetota* was found only in Przewalski’s gazelle (prTN). At the family level, 52 of the 71 abundant families were found in all 6 populations, *WCHB1-41*, *Planococcaceae*, *Bradymonadales*, *Bogoriellaceae*, and *Microbacteriaceae* were found exclusively in Przewalski’s gazelle. Among the 135 rare families, 47 were found in all populations (34.8%). Among those taxa, 20% of rare families were endemic to Przewalski’s gazelle, while only 5.9% were endemic to Tibetan gazelle.

#### *α*-Diversity

**Figure 3:**
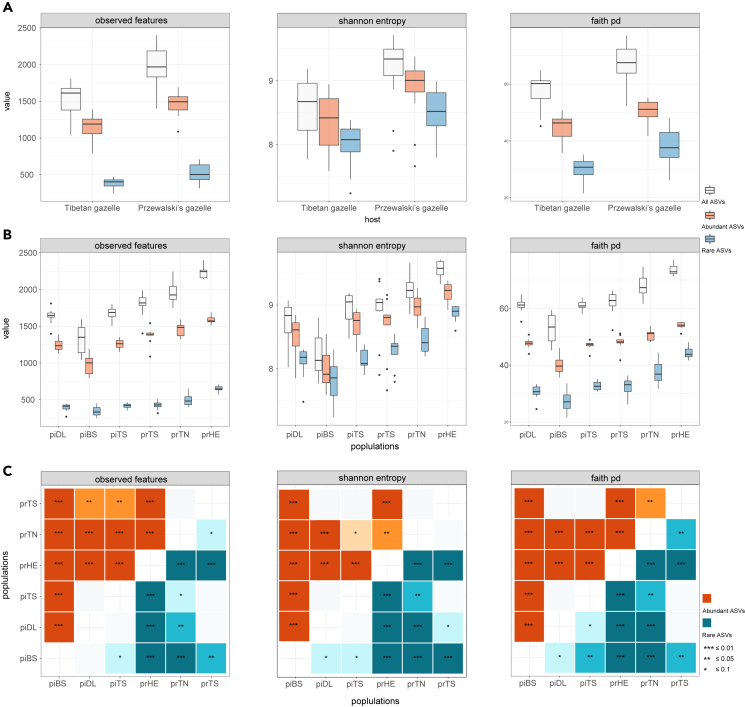
Comparison of *α*-diversity in gut microbiota among gazelle populations *α*-diversity (all ASVs, abundant ASVs, and rare ASVs) varied significantly by (A) species, and by (B) populations. (C) 73.3%–93.3% of populations differed significantly in the *α*-diversity of abundant ASVs and rare ASVs (pairwise Wilcoxon rank-sum test with a 10% false discovery rate). See also Tables S5 and S6.

*α*-diversity differed significantly between two related species and varied dramatically across all populations. In total, the *S*_*obs*_, Shannon and Faith’s phylogenetic diversity (PD) indices of abundant ASVs from these two species were significantly higher than rare ASVs (Kruskal-Wallis rank-sum test; *S*_*obs*_: *H* = 84.76; Shannon: *H* = 17.80; PD: *H* = 68.60; p < 0.001; Figure 3A). This suggested that, overall, the *α*-diversity of abundant bacterial communities in Tibetan gazelle and Przewalski’s gazelle was higher than that of rare bacterial taxa. Furthermore, Przewalski’s gazelle showed significantly higher *α*-diversity indices in all ASVs, abundant ASVs, and rare ASVs than Tibetan gazelle (Kruskal-Wallis rank-sum test, p < 0.001; Figure 3B; Table S5). The additional pairwise comparison shows that most population pairs (73.3%–93.3%) differed significantly in the *α*-diversity of abundant ASVs and rare ASVs after correcting for multiple testing. (Pairwise Wilcoxon rank-sum test, *adjusted* p *values* < 0.1; Figure 3C).

#### *β*-Diversity

**Figure 4:**
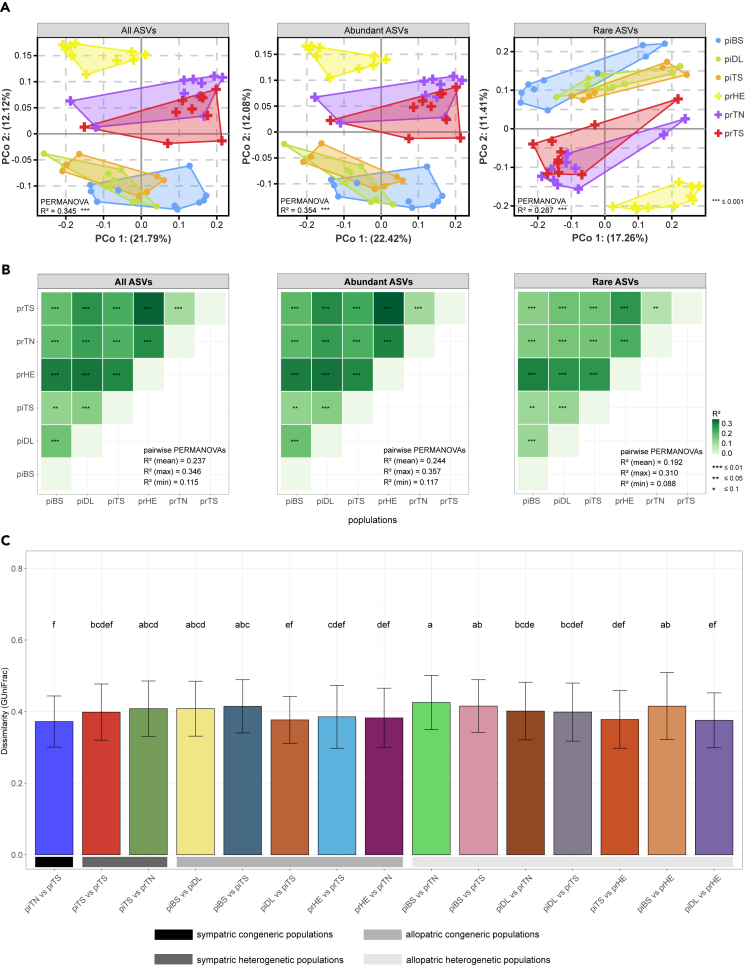
Sympatric populations harbor distinct gut microbiota (A) Principal coordinates analysis (PCoA) showing axes 1 and 2 of individual gut microbiota, colored by populations. (B) All populations differed significantly in microbiota composition (pairwise PERMANOVAs). (C) The sympatric Tibetan gazelle and Przewalski’s gazelle gut microbiota did not show significant convergence. Individuals’ gut microbiota from different pairs of host populations is shown as the average pairwise Generalized UniFrac dissimilarities in each bar (all ASVs), data are represented as mean ± SD. The post hoc test used Fisher’s least significant difference (LSD) test with Bonferroni correction (*adjusted* p *values* < 0.05). See also Table S8 and Figure S5.

Population membership explained 34.5%, 35.4%, and 28.7% (PERMANOVA; p < 0.0001) of the variation in microbial *β*-diversity of all ASVs, abundant ASVs, and rare ASVs, respectively (Figure 4A). Besides, all population pairs differed significantly in microbial composition after correcting for multiple testing (*adjusted* p *values* < 0.1 of significant pairwise PERMANOVAs; Figure 4B).

In summary, gut microbiota composition and diversity exhibited significant differences not only between Tibetan and Przewalski’s gazelles but also within their geographically distinct populations. These variations suggest a notable influence of both host species and environmental factors associated with spatial isolation on gut microbiota across different species and populations.

### Gut microbiota of sympatric populations did not show significant convergence

To investigate the potential for environmental factors to drive convergence in gut microbiota among closely related species in shared habitats, we further analyzed whether sympatric distribution leads to significant convergence in gut microbiota. The sympatric populations (piTS, prTS, and prTN) showed no obvious convergence compared with allopatric populations in microbiota composition, regardless of abundant or rare taxa (Figure 2D). Additionally, no taxonomic groups were exclusively present in sympatric populations but absent in all allopatric populations, across both abundant and rare taxa (Figure S4).

To determine the influence of sympatric distribution on the *α*-diversity of gut microbiota among related species, we employed Fisher’s exact test of independence and a generalized linear mixed model (GLMM). Fisher’s test assessed whether the significant differences in *α*-diversity of all ASVs, abundant ASVs, and rare ASVs were related to sympatric distribution, yielding a p *value* greater than 0.05, indicating no significant relationship (Table S6). Additionally, the GLMM analysis, which included population membership as a random effect, was utilized to explore if host identity or sympatry could predict the *α*-diversity across all types of ASVs (Table S7). The results demonstrated that sympatric distribution did not significantly predict *α*-diversity, for all, abundant, or rare ASVs.

Similar to *α*-diversity, there was no significant convergence between closely related species due to the sympatric distribution. Fisher’s exact test of independence indicated that the significant difference in *β*-diversity of all-, abundant-, and rare ASVs were not related to the sympatry (p = 1, Table S8). Furthermore, pairwise GuniFrac dissimilarities indicated that the gut microbiota of sympatric heterogenetic populations did not exhibit greater similarity to the gut microbiota of allopatric congeneric populations, or allopatric heterogenetic populations, regardless of abundant ASVs or rare ASVs (Kruskal-Wallis rank-sum test, post hoc test is using fisher’s least significant difference (LSD) test with Bonferroni correction, *adjusted* p *values* < 0.05; Figures 4C and S5).

### Covariation of spatial factors and soil-associated microbes to Przewalski’s gazelle gut microbiota

Given our previous findings of interspecies and interpopulation gut microbiota differences without convergence, we hypothesized that the relationship between gut microbiota composition and external environmental changes might differ between Tibetan and Przewalski’s gazelles due to species-specific characteristics. To investigate this, we first analyzed the proportion of soil-associated microbes (PSM) in the gut microbiota. PSM refers to the microbial taxa present in the soil environment that are potentially ingested and incorporated into the gazelles’ gut microbiota, quantified using the fast expectation maximization microbial source tracking (FEAST) method.^62^ Overall, the PSM was relatively low in both Tibetan gazelle (0.75% ± 0.60%) and Przewalski’s gazelle (3.15% ± 4.94%). However, PSM was significantly higher in Przewalski’s gazelle compared to Tibetan gazelle (Kruskal-Wallis rank-sum test, *H =* 3.8644, df = 1, p = 0.04932). Moreover, these differences persisted across 6 populations (Kruskal-Wallis rank-sum test, *H* = 37.356, df = 5, p < 0.0001). Pairwise comparisons showed that except for prHE Vs. piBS and prTN vs. piTS, PSM differed significantly between all other population pairs (Pairwise Wilcoxon rank-sum test, *adjusted* p *values* < 0.1; Table S9). Furthermore, Fisher’s exact test of independence shows that the significant differences in PSM were also not related to the sympatry (p = 0.3714). Finally, the PERMANOVA was performed to investigate the relative effect on the variation of PSM. The host identity and population membership explained 42.9% of the variation of PSM (PERMANOVA, p = 0.0001), and the effect of sympatry was minor and insignificant. To determine the relationship between PSM and the gut microbiota of two closely related species, we performed Mantel tests for each species, respectively. The results demonstrated a significant correlation between PSM and overall gut microbiota composition in Przewalski’s gazelle, including all ASVs (r = 0.212, p = 0.0036), abundant ASVs (r = 0.217, p = 0.0043) and rare ASVs (r = 0.222, p = 0.0031), however, no such correlation was observed in Tibetan gazelle. Suggest potential species-specific interactions of gut microbiota with environmental microbes in these two gazelle species.

In addition, both spatial factors, geographical distance, and PSM could potentially influence the *β*-diversity of gut microbiota in Tibetan gazelle and Przewalski’s gazelle. To discern the influence of those factors on the gut microbiota *β*-diversity of Tibetan gazelle and Przewalski’s gazelle, partial Mantel test analysis was performed, which allowed us to examine the influence of one factor while controlling for the others. When controlling for the spatial factors (PC1) and geographic distance, PSM remained significantly correlated with gut microbiota dissimilarity of Przewalski’s gazelle. In contrast, when controlling for the PSM and geographic distance, the spatial factors (PC1) were significantly correlated with Przewalski’s gazelle gut microbiota dissimilarity. Notably, there were no factors significantly associated with the gut microbiota dissimilarity of Tibetan gazelle (Table 1). The multiple regression on (dis)similarity matrices analysis (MRM) was used to further identify the relative contributions of spatial factors (PC1) and PSM to gut microbiota dissimilarity of Przewalski’s gazelle. The MRM model explained 30.4%, 31.8%, and 22.8% (MRM, p < 0.0001) of the variation in Przewalski’s gazelle gut microbiota dissimilarity of all-, abundant-, and rare ASVs, respectively. In each MRM model, the effect of spatial factors (PC1) was all greater than that of PSM (Table 2).

**Table 1:** The results of Spearman correlation between the bacterial community distances and the proportions of soil-associated microbes distance (PSM), spatial factors (PC1) distance or geographic distance for pairwise samples of Tibetan gazelle (n = 27) and Przewalski’s gazelle (n = 30) using partial Mantel test

Correlation between bacterial community distance and	Controlling for:	Tibetan gazelle						Przewalski’s gazelle					
		All ASVs		Abundant ASVs		Rare ASVs		All ASVs		Abundant ASVs		Rare ASVs	
		*R*	*p*	*R*	*p*	*R*	*p*	*R*	*p*	*R*	*p*	*R*	*p*
PSM^a^	SF + G	0.164	0.222	0.052	0.239	0.038	0.268	0.164	0.018	0.168	0.016	0.181	0.009
SF^b^	PSM + G	0.106	0.061	0.105	0.063	0.083	0.085	0.214	0.002	0.210	0.002	0.179	0.004
G^c^	PSM + SF	−0.061	0.808	−0.059	0.803	−0.016	0.595	−0.018	0.624	−0.006	0.549	−0.024	0.679

*p* values are one-tailed tests based on 9,999 permutations.

a The proportions of soil-associated microbes in the gut microbiota of Przewalski’s gazelle distance.

b The spatial factors (PC1) distance.

c The geographic distance.

**Table 2:** The results of the multiple regression analysis on matrices analysis for gut microbiota of the Przewalski’s gazelle

	All ASVs		Abundant ASVs		Rare ASVs	
	*R*^*2*^ = 0.3040^a^	p < 0.0001	*R*^*2*^ = 0.3184	p < 0.0001	*R*^*2*^ = 0.2279	p < 0.0001
	*b*	*P*	*b*	*p*	*b*	*p*
PSM^b^	0.14	0.0221	0.14	0.0197	0.16	0.0001
SF^c^	0.51	0.0001	0.53	0.0001	0.43	0.0001

a The variation (*R*^*2*^) of community dissimilarity (all-, abundant-, or rare ASVs) that is explained by the remaining variables. The partial regression coefficients (*b*) and associated p values are reported from permutation test (nperm = 9,999).

b The proportions of soil-associated microbes in the gut microbiota of Przewalski’s Gazelles distance.

c The spatial factors (PC1) distance.

Overall, these results support the hypothesis that the relationship between gut microbiota composition and external environmental changes varies between species, highlighting the influence of host-specific differences.

### Ecological assembly mechanism differs between the Tibetan gazelle and Przewalski’s gazelle

**Figure 5:**
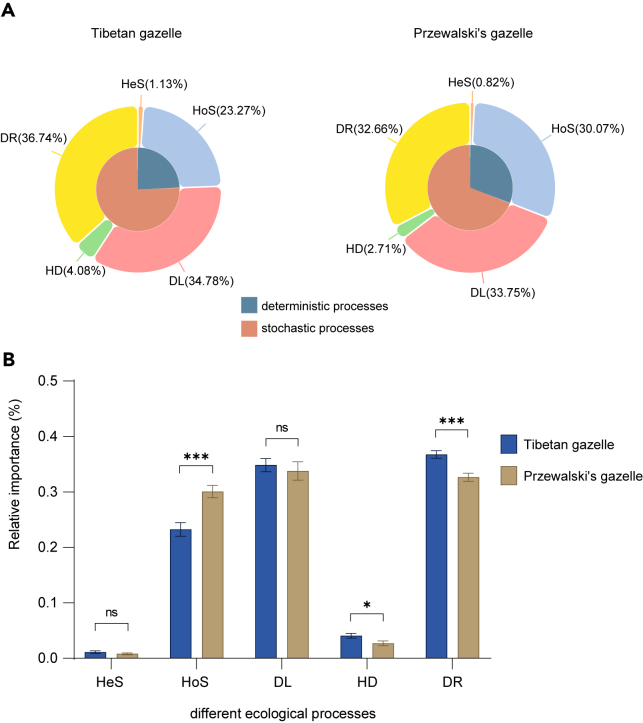
The gut microbiota assembly process of Przewalski’s gazelle was shifted toward homogeneous selection relative to that of the Tibetan gazelle (A) The relative importance of the different ecological processes in bacterial assembly for Tibetan gazelle and Przewalski’s gazelle. Dispersal limitation (DL); drift (DR); homogenizing dispersal (HD), heterogenous selection (HeS), and homogenous selection (HoS). (B) Changes of HoS, DR, and HD between Tibetan gazelle and Przewalski’s gazelle, data are represented as mean ± SD. One-side significance based on bootstrapping test was expressed as ∗∗∗p < 0.001; ∗∗p < 0.01; ∗p < 0.05.

To elucidate the ecological assembly mechanisms underlying the distinct gut microbiota profiles of the two gazelle species, we quantified their gut microbiota assembly processes. The gut microbiota ecological assembly process of Tibetan gazelle and Przewalski’s gazelle were mainly dominated by homogeneous selection (HoS, refers to the scenario where the gut environment within hosts of a species leads to the selection of similar microbial communities; 23.3%–30.1%), dispersal limitation (DL, refers to the restriction in microbial exchange between individual gazelles of the same species due to physical separation; 33.8%–34.8%), and random drift (DR, refers to the stochastic changes in the microbial communities within hosts of a species; 32.7%–36.7%). HoS (*Cohen.d* = −5.80, p < 0.0001) is more important in Przewalski’s gazelle than in Tibetan gazelle, meanwhile, the proportion of HD (*Cohen.d* = 0.336, p = 0.0142) and DR (*Cohen.d* = 5.77, p < 0.0001) processes decreased. This suggests that the gut microbiota assembly process of Przewalski’s gazelle was shifted toward HoS relative to that of the Tibetan gazelle (Figures 5A and 5B).

**Figure 6:**
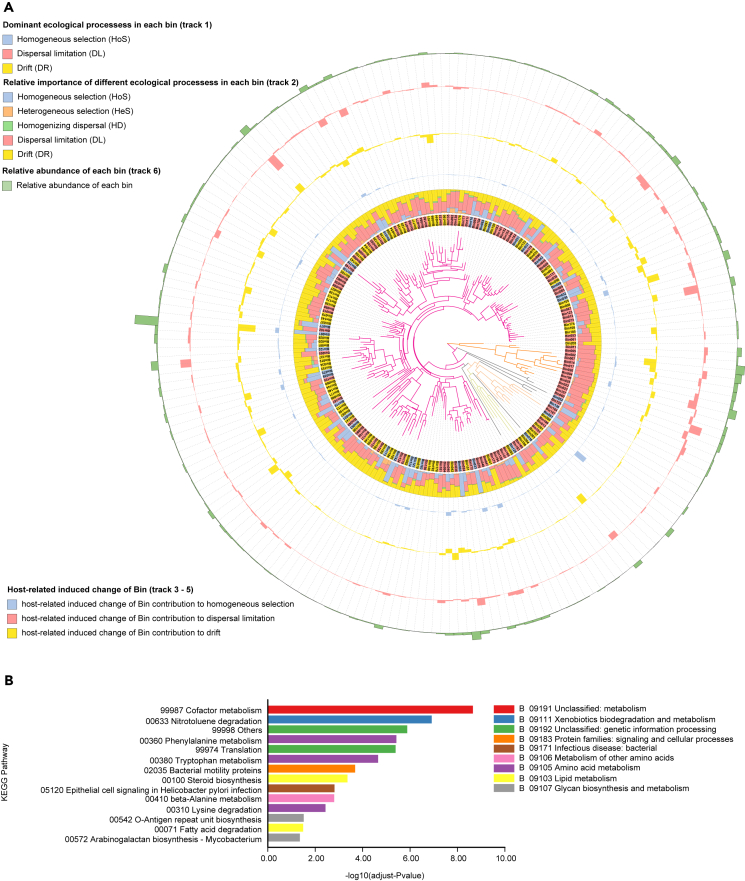
Gut microbiota assembly patterns and functions prediction (A) Assembly mechanisms across different phylogenetic bins for both Tibetan gazelle and Przewalski’s gazelle. A phylogenetic tree was displayed at the center and colored by phylum, representing the relationship of the top ASVs of each bin. Tracks from the inner to the outer layer of the plot represent the dominant ecological process of each bin for both Tibetan gazelle and Przewalski’s gazelle (track 1, colored by the dominant ecological process), the relative importance of different ecological processes in each bin (track 2, presented as stacked bar-plots), host-induced change in bin contribution to HoS (track 3), DR (track 4), DL (track 5), and the relative abundance of each of the bins (track 6). (B) The pathways enrichment of those ASVs that contributed other processes (DR, DL, HD, and HeS) in Tibetan gazelle but HoS in Przewalski’s gazelle. See also Tables S10, S11, and S12.

The iCAMP allocated the 5,347 observed ASVs into 205 phylogenetic bins, each of which was analyzed separately as shown in Figure 6A. The results revealed that HoS dominated the ecological assembly process of 32 bins (15.6% of the bin and 26.1% of relative abundance), mainly from *Oscillospiraceae*, *Christensenellaceae*, and *Lachnospiraceae*. The relative abundances of those three families accounted for 34.2%, 12.2%, and 13.1% of all HoS dominant bins, respectively. In addition, 78 bins (38.1% of the bin and 23.8% of relative abundance) were dominated by DR, mainly from *Oscillospiraceae* (18.2%), *Lachnospiraceae* (15.3%), *Monoglobaceae* (12.7%), and *Oscillospirales UCG-010* (10.5%). Then, 95 bins (46.3% of bin and 50.1% of relative abundance) were dominated by DL, mainly from *Lachnospiraceae* (15.4%) and *Rikenellaceae* (10.4%) (Tables S10 andS11).

The bins contributing to other processes (DR, DL, HD, or HeS) in Tibetan gazelle but HoS in Przewalski’s gazelle (Table S12) was further analyzed. A total of 15 bins (containing 406 ASVs) were changed, comprising 8.6% of the total abundance, mainly belonged to *Oscillospiraceae* (18.2%), *[Eubacterium] coprostanoligenes group* (18.0%), *Christensenellaceae* (17.0%), *Akkermansiaceae* (12.5%), and *Lachnospiraceae* (12.4%). The KEGG pathway enrichment analysis of these ASVs showed that the putative functions were mainly related to macronutrient and micronutrient metabolism (Figure 6B).

## Discussion

### Differences in intrinsic adaptability and adaptive potential prevent convergence of gut microbiota in sympatric closely related species

In our study, sympatric populations of Tibetan gazelle and Przewalski’s gazelle did not exhibit significant convergence in gut microbiota. This lack of convergence was observed across all ASVs, abundant ASVs, and rare ASVs, despite sharing a common environment. Moreover, despite shared exposure to environmental microbes such as soil microbes, we observed no convergence in gut microbiota. This suggests that differences in the intrinsic adaptability and adaptive potential of Tibetan gazelle and Przewalski’s gazelle play a more substantial role in shaping their gut microbiota, counteracting the homogenizing effect of a shared environment.

In closely related host species, the external environment is a dominant driver of gut microbial variation.^6^ Our findings seem to contradict this general view. Investigations of baboons in hybrid zones reveal that the host genetic background fails to predict baboons gut microbiota effectively.^6^ However, it is essential to notice that genetic differences between two closely related species not necessarily reflect differences in their intrinsic adaptability and potential for adaptability. In some cases, genetic differences between closely related species may represent neutral or nearly neutral changes that have little impact on their adaptability or adaptive potential.^30^ Additionally, hybridization between closely related species can have complex and context-dependent effects on the intrinsic adaptability and adaptive potential of the resulting hybrid individuals. The outcomes of hybridization may range from reduced fitness due to outbreeding depression to increased fitness through the introduction of novel genetic variation and the potential for adaptation to new environments or ecological niches.^31^ This highlights the importance of considering the unique intrinsic adaptability and adaptive potential of each species when examining the factors that shape their gut microbiota.

### Variation in gut microbiota-environment interactions influenced by host intrinsic adaptability and adaptive potential

Our study reveals divergent gut microbiota-environment interactions between Przewalski’s gazelle and Tibetan gazelle, likely attributable to differences in their intrinsic adaptability and adaptive potential. Potential habitat assessments grounded in niche theory offer a proxy for niche breadth, broader potential habitat coverage generally suggests a wider realized niche,^32^ which corresponds to greater adaptability of those persisting species.^33^ The potentially suitable habitat area for Przewalski’s gazelle is only 3.6% of the Qinghai-Tibet Plateau, whereas Tibetan gazelle occupies 37.1%. This nearly 10-fold difference in inhabitable range indicates significantly lower intrinsic adaptability in Przewalski’s gazelle. Moreover, Przewalski’s gazelle has historically experienced a population bottleneck, leading to a significant decline in its population and a marked reduction in genetic diversity compared to Tibetan gazelle.^17^^,^^18^ Genetic diversity analysis shows that nucleotide diversity (π) of Przewalski’s gazelle mitochondrial control region is 0.0047,^34^ markedly lower compared to 0.1004 in Tibetan gazelle.^35^ Since low genetic diversity compromises adaptive potential,^36^^,^^37^ this further suggests that the adaptive potential of Przewalski’s gazelle is lower than that of Tibetan gazelle. We observed distinct disparities in gut microbiota composition and diversity between two closely related species, Przewalski’s gazelle and Tibetan gazelle, with Przewalski’s gazelle exhibiting a more pronounced response to environmental changes.

In microbial communities associated with hosts, host filtering refers to the process by which only certain microbial taxa are able to colonize or persist within the host’s environment. This selection process is important in addition to the biotic interactions that occur among gut microbiota.^38^ If the abiotic and biotic environmental conditions are uniform, there will be little variation in the community structure or compositional turnover, which is known as homogeneous selection (HoS).^26^ Therefore, the magnitude of HoS in the assembly of gut microbiota not only reflects the specificity of the within-host environment when compared with other species but also highlights the degree of homogeneity among individuals within a single species. In our study, the gut microbial community assembly processes of Przewalski’s gazelle were shifted toward HoS, suggesting that, across various environments, individuals within the Przewalski’s gazelle population share a more specialized reliance on the “ecosystem services” provided by the gut microbiota to adapt to environmental changes, emphasizing the unique, collective needs of this species in comparison to Tibetan gazelle.

In Przewalski’s gazelle, with lower intrinsic adaptability, the gut microbiota appears to play a crucial role in adapting to environmental shifts, by providing a broader range of ecological functions and services, contributing to the host’s ability to adjust to changing conditions. In contrast, Tibetan gazelle, with its stronger intrinsic adaptability and adaptive potential, seems to rely less on gut microbiota composition and functionality for adapting to different environments at the scale of our study. The observed variation in gut microbiota-environment interactions between the two species underscores the importance of considering host adaptability and adaptive potential when investigating host-microbiome dynamics. Furthermore, this implies that species with greater adaptability and adaptive potential may have different strategies for managing their gut microbiota in response to environmental challenges. If this phenomenon is prevalent, it could have significant implications for our understanding of the co-evolutionary dynamics between hosts and their gut microbiota.^1^

### Specific gut microbiota requirements in Przewalski’s gazelle

The external ecology of the host influences its internal ecology, which has implications for the “ecosystem services” that microbiomes provide.^6^ In the case of Przewalski’s gazelle, this heightened sensitivity reflects their need for specific gut microbiota composition and functionality to cope with environmental changes. The taxa that contributed other processes (DR, DL, HD, or HeS) in Tibetan gazelle but HoS in Przewalski’s gazelle mainly belonged to *Oscillospiraceae*, *[Eubacterium] coprostanoligenes group*, *Christensenellaceae*, *Akkermansiaceae*, and *Lachnospiraceae*. The KEGG pathway enrichment of those ASVs showed that the functions were mainly related to the metabolism of micronutrients and macronutrients, including aromatic amino acids like phenylalanine and tryptophan, which influence the host’s immune function and health.^39^ Additionally, arabinogalactan can improve the gut barrier function,^40^ and cofactors and fatty acids have a wide range of effects on the host and the gut microbial community.^41^^,^^42^ Exposure to nitrotoluene can detrimentally modify the microbiome into a pro-inflammatory state.^43^ Taken together, the increased abundance of microbes associated with key pathways related to immunity, health maintenance, and homeostasis regulation implies that Przewalski’s gazelle relies more on the “ecosystem services” of gut microbiota to adapt to varying environments and satisfy their specific nutritional requirements, compared to Tibetan gazelle. By selectively enriching such beneficial microbes, Przewalski’s gazelle exhibits a stronger dependence on gut microbiota to maintain homeostasis for adaptation.

Our results showed that the *α*-diversity of gut microbiota was significantly higher in Przewalski’s Gazelles than in Tibetan gazelles. The higher *α*-diversity of gut microbiota could mean that the functionality, stability, and eco-services of the community were also improved.^44^^,^^45^ Moreover, the gut microbial generation time is much shorter than that of the host, which allows for genetic evolution over shorter periods, and thus might play a critical role when vertebrates acclimate and adapt to environmental change.^46^ Our results suggest that maintaining a relatively higher *α*-diversity of gut microbiota may be essential to Przewalski’s gazelle, offering not only better eco-services and functions but also resistance to the invasion by external microbes, also known as “insurance effect”.^47^ However, maintaining a high *α*-diversity comes at a cost, as more abundant gut microbes also mean a higher demand for the total energy intake, not only to support the host’s own needs but also to satisfy the gut microbiota. From this perspective, for Tibetan gazelle, which is more ecologically adaptive, maintaining a relatively lower *α*-diversity gut microbiota may be an advantageous alternative.

In addition, our results reveal that a certain proportion of the gut microbiota in both gazelle species originates from soil microbial communities. Moreover, there is significant variation between the populations, which is consistent with previous evidence on environment-specific microbial acquisition.^12^^,^^48^^,^^49^ Notably, Przewalski’s gazelles exhibit a higher PSM compared to Tibetan gazelles. Furthermore, a significant correlation was found between PSM and the gut microbiota composition in Przewalski’s gazelles, a pattern absent in Tibetan gazelles. This suggests the gut microbiota composition in Przewalski’s gazelles may be influenced by specific environmental acquisitions, as opposed to the more random pattern seen in Tibetan gazelles. The observed pattern in Przewalski’s gazelles raises two possibilities: the gazelles might be selectively enriching specific soil microbes within their gut microbiota as an adaptive response to environmental challenges,^50^ or the higher PSM might indicate a differential microbial colonization resistance mechanism, potentially due to their lower adaptability. However, given the overall low proportion of PSM in both species, the latter explanation might align more closely with our findings. Further research is needed to elucidate the specific mechanisms underlying these gut-soil microbe dynamics and how they relate to the host’s adaptability and environmental interactions.

In summary, our study highlights the importance of intrinsic adaptability and adaptive potential in shaping gut microbiota composition and functionality among closely related sympatric species. We demonstrate that differences in these factors can lead to distinct gut microbiota-environment interactions and unique gut microbial community assembly processes. This finding emphasizes the need to consider host adaptability and adaptive potential when investigating host-microbiome dynamics and co-evolutionary relationships. Assessing a species’ intrinsic adaptability and adaptive potential is of paramount importance in conservation biology, as it is integral to endangered species research and provides a foundation for the implementation of effective conservation measures. Our study offers a practical approach and theoretical framework to address these challenges.

### Limitations of the study

Unfortunately, in our qualitative research, we were unable to further distinguish and elucidate the independent relationships of gut microbiota with the host’s intrinsic adaptability and with the host’s adaptive potential, respectively. Future research should focus on this area to address several key scientific questions that remain unanswered.

## STAR★Methods

### Key resources table

**Table:** 

REAGENT or RESOURCE	SOURCE	IDENTIFIER
**Biological samples**		

Fecal and soil samples from different populations of Tibetan gazelle and Przewalski’s gazelle and their habitats were used for microbiome analysis	This paper	N/A

**Chemicals, peptides, and recombinant proteins**		

E.Z.N.A.® soil DNA Kit	Omega Bio-Tek	Cat# D5625-02
TransStart® FastPfu DNA Polymerase	TransGen Biotech	Cat# AP221-01
AxyPrep™ DNA Gel Extraction Kit	Axygen Scientific	Cat# AP-96-GX-24

**Deposited data**		

Raw sequencing data	This paper	BioProject: PRJNA902834
16S SILVA database (version 138.1)	Quast et al.^51^	https://www.arb-silva.de/; RRID:SCR_006423
KEGG database	Kanehisa et al.^52^	https://www.kegg.jp/; RRID:SCR_012773

**Experimental models: Organisms/strains**		

Tibetan gazelle (Procapra picticaudata)	This paper	RRID:NCBITaxon_59540
Przewalski’s gazelle (Procapra przewalskii)	This paper	NCBI:txid157668

**Oligonucleotides**		

338F (5′-ACTCCTACGGGAGGCAGCAG-3′)	Fierer et al.^53^	N/A
806R (5′-GGACTACHVGGGTWTCTAAT-3′)	Caporaso et al.^54^	N/A

**Software and algorithms**		

QIIME2-2022.2	Bolyen et al.^55^	https://qiime2.org/; RRID:SCR_021258
DADA2	Callahan et al.^56^	https://benjjneb.github.io/dada2/; RRID:SCR_023519
Figaro	Weinstein et al.^57^	https://github.com/Zymo-Research/figaro
RESCRIPt	Robeson et al.^58^	https://github.com/bokulich-lab/RESCRIPt
q2-feature-classifier plugin	Bokulich et al.^59^	https://github.com/qiime2/q2-feature-classifier
MAFFT	Katoh et al.^60^	https://mafft.cbrc.jp/alignment/software/; RRID:SCR_011811
FastTree 2	Price et al.^61^	http://meta.microbesonline.org/fasttree/; RRID:SCR_015501
FEAST package	Shenhav et al.^62^	https://github.com/cozygene/FEAST
MultiCoLA package	Gobet et al.^63^	https://www.mpi-bremen.de/Software-2.html#section1550
geosphere package	Hijmans et al.^64^	https://github.com/rspatial/geosphere
vegan package	Oksanen et al.^65^	https://vegandevs.github.io/vegan/; RRID:SCR_011950
R Project for Statistical Computing	R Core Team.^66^	https://www.r-project.org/; RRID:SCR_001905
Picante package	Kembel et al.^67^	https://github.com/skembel/picante
ggplot2 package	Wickham et al.^68^	https://ggplot2.tidyverse.org/; RRID:SCR_014601
lme4 package	Bates et al.^69^	https://github.com/lme4/lme4; RRID:SCR_015654
ecodist package	Goslee et al.^70^	https://github.com/phiala/ecodist
iCAMP package	Ning et al.^71^	https://github.com/DaliangNing/iCAMP1
PICRUSt2 package	Douglas et al.^72^	https://github.com/picrust/picrust2; RRID:SCR_022647
TBtools	Chen et al.^73^	https://github.com/CJ-Chen/TBtools-II; RRID:SCR_023018
Rstudio	RStudio Team.^74^	https://posit.co/; RRID:SCR_000432

**Other**		

NanoDrop™ 2000 UV-vis Spectrophotometer	Thermo Fisher Scientific	RRID:SCR_018042
GeneAmp™ PCR System 9700	Applied Biosystems	RRID:SCR_018436
Quantus™ Fluorometer	Promega	N/A
Illumina MiSeq sequencing platform	Illumina	RRID:SCR_016379

### Resource availability

#### Lead contact

Further information and requests should be directed to and will be fulfilled by the lead contact, Prof. Tongzuo Zhang (zhangtz@nwipb.cas.cn).

#### Materials availability

This study did not generate new unique reagents.

#### Data and code availability

• The raw 16S rRNA sequencing data of this article has been deposited in the National Center for Biotechnology Information (NCBI) database. Accession number is listed in the key resources table. Full metadata is available in Table S1.
• This paper does not report any original code.
• Any additional information required to reanalyze the data reported in this paper is available from the lead contact upon request.

### Experimental model and study participant details

#### Ethics statement

All experiments, including the sample collection methods, followed the principles of the Ethical Committee for Experimental Animal Welfare of the Northwest Institute of Plateau Biology.

#### Animals

Fecal samples from Tibetan gazelle and Przewalski’s gazelle were collected from 6 distinct populations in Qinghai province, China. Two populations of Przewalski’s gazelle (prTN, n = 10; prTS, n = 10) and one population of Tibetan gazelle (piTS, n = 7) were collected from the Shengge Area upon the Upper Buha River in Tianjun County, where Tibetan gazelle and Przewalski’s gazelle coexisted. Fecal samples from populations of Tibetan gazelle (piDL, n = 10; piBS, n = 10) and Przewalski’s gazelle (prHE, n = 10) were collected from areas with a similar geographic distance to the sympatric area. Soil samples corresponding to each population were also collected to represent the environmental microbiota of each site (Figure 1). All samples were collected from 4 December to 11 December 2021, to avoid temporal variation (Table S1).

### Method details

All fecal samples in Tibetan gazelle and Przewalski’s gazelle were collected passively through observation and tracking, with samples being collected as soon as animals defecated and moved away. A minimum of 8 moist, soil-free fecal pellets were collected from each individual. Moreover, before collecting soil samples, areas with animal defecation were avoided, and instead, feeding areas were identified to prevent possible contamination of topsoil by feces. Within those areas, the three 3 m × 3 m quadrats were demarcated and then soil samples from each quadrat (the vertex and center) were collected. For each quadrat, we thoroughly mixed five aliquots of topsoil (top 2–5 cm) and then sieved mixture through a 0.4 mm mesh to remove rocks and roots. After mixing, a total of 3 soil samples were obtained from each quadrat, which were used to represent the soil microbial community at the sampling site.

During sample collection, disposable polyethylene (PE) gloves were used to prevent contamination and were replaced between samples. All samples were first placed in self-sealing bags, then covered by aluminum foil and subsequently stored in a portable liquid nitrogen tank and shipped to the laboratory. The samples were preserved at −80°C in the Northwest Institute of Plateau Biology, Chinese Academy of Sciences.

### Quantification and statistical analysis

#### DNA extraction, PCR amplification, and sequencing

The total genomic DNA of each sample was extracted from a 500 mg well-mixed sample using the E.Z.N.A. soil DNA Kit (Omega Bio-Tek, Norcross, GA, U.S.) following the manufacturer’s instructions. DNA degradation was monitored by analyzing 1% agarose gels, and the purity and concentration of the DNA were determined using a NanoDrop 2000 UV-vis spectrophotometer (Thermo Scientific, Wilmington, USA). The hypervariable V3-V4 region of bacterial 16S rRNA gene were subsequently amplified in triplicate using 338F–806R specific primers (5′-ACTCCTACGGGAGGCAGCAG-3′, 806R: 5′-GGACTACHVGGGTWTCTAAT-3').^53^^,^^54^

The PCR amplification of the 16S rRNA gene was performed in triplicate using an ABI GeneAmp 9700 PCR thermocycler (ABI, CA, USA) in a 20 μL mixtures, containing 5 × TransStart FastPfu buffer 4 μL, 2.5 mM dNTPs 2 μL, forward primer (5 μM) 0.8 μL, reverse primer (5 μM) 0.8 μL, BSA 0.2 μL, TransStart FastPfu DNA Polymerase 0.4 μL,10 ng template DNA, and ddH_2_O up to 20 μL. The thermal cycling procedure was as follows: initial denaturation at 95°C for 3 min, followed by 27 cycles of denaturing at 95°C for 30 s, annealing at 55°C for 30 s and extension at 72°C for 45 s, and a final extension at 72°C for 10 min, end at 10°C. The PCR products of each sample were then mixed, assessed by 2% agarose gel electrophoresis and purified using the AxyPrep DNA Gel Extraction Kit (Axygen Biosciences, Union City, CA, USA) according to the manufacturer’s instructions and quantified using Quantus Fluorometer (Promega, USA).

Purified amplicons were then pooled equimolarly, and paired-end sequenced (2 × 300) on an Illumina MiSeq sequencing platform (Illumina, San Diego, USA) according to the standard protocols by Majorbio Bio-Pharm Technology Co. Ltd. (Shanghai, China), yielding 16,483,828 unpaired raw reads.

#### Bioinformatics analysis of sequences

The 16S rRNA gene sequence analysis was mainly conducted in QIIME2-2022.2 (Figure S1).^55^ The raw sequence data were demultiplexed based on barcode sequences using the q2-demux plugin, followed by trimming adapter and primer sequences with the q2-cutadapt plugin.^75^ DADA2 was used (via q2-dada2 plugin) to denoise the sequences, which included remove low-quality and chimeric sequences, error correction, and merging of paired-end reads.^56^ The optimized truncation length parameters were accessed using Figaro.^57^ Finally, a raw ASVs table and ASVs representative sequence were generated.

Taxonomic profiling studies rely on high-quality sequence taxonomy reference databases, to improve taxonomic classification, the reference sequence annotation and curation pipeline (RESCRIPt) was used to prepare a QIIME2 compatible SILVA 16S rRNA gene taxonomy classifier,^58^ based on the SILVA curated NR99 (version 138.1) SSU database^51^ following the tutorial suggested by the authors (https://forum.qiime2.org/t/processing-filtering-and-evaluating-the-silva-database-and-other-reference-sequence-data-with-rescript/15494). The V3-V4 region of the 16S RNA gene was extracted from the SILVA SSU database and used to train an amplicon-region specific naive-Bayes classifier, which allows for more robust taxonomic classification. Taxonomy classification of ASVs was done via Q2-feature-classifier classify-sklearn with a 0.8 confidence threshold.^59^ Then, taxonomy-based filtering was applied to remove all ASVs classified as mitochondria, chloroplasts, or archaea. Any ASVs that appeared in less than 10% of the total samples or had a percent relative abundance lower than 0.0001% were also filtered out.

The align-to-tree-mafft-fasttree pipeline from the q2-phylogeny plugin was adopted to generate a rooted phylogenetic tree, which was used for calculating Faith’s Phylogenetic Diversity^76^ and Generalized UniFrac distances.^77^ To achieve this, the pipeline utilized the mafft program^60^ to perform a multiple sequence alignment of the representative sequences, followed by masking the alignment to remove highly variable positions. FastTree^61^ was then used to generate an unrooted phylogenetic tree from the masked alignment. Finally, the root of the tree was placed using midpoint rooting to generate a rooted tree.

To determine if the richness of all samples had been fully observed, an alpha rarefaction curve (iterations = 999, steps = 10) was constructed using q2-diversity (alpha-rarefaction). The ASV table was then rarefied to the lowest sequencing depth of all samples (26,685 sequences) to remove sample heterogeneity and for all further analyses except FEAST analysis, which used the unrarefied ASVs table.

#### Assessing the contribution of the soil microbiota to the gazelle fecal microbiota

Fast expectation maximization microbial source tracking (FEAST) was designed to unravel the origins of microbial communities by assuming each ‘Sink’ sample is a convex combination of known and unknown ‘Sources’ and uses multinomial distributions and machine-learning classification to model the microbial source tracking.^62^ FEAST was adopted to reveal the proportions of soil-associated microbes (PSM) in the gazelle fecal microbiota. In this analysis, the soil samples collected in a given site (n = 3) represented the potential exposure to environmental microbes and are categorized as ‘Sources’, and the corresponding fecal samples in this given site are categorized as ‘Sink’, thereby providing insights into the extent of environmental microbial influence on the gut microbiota. Note, the raw ASVs table was used for the FEAST analysis with the following parameters: COVERAGE = 26,685 and EM_iterations = 10,000,000.

#### Multivariate cutoff level analysis to define abundant and rare taxa

Multivariate cutoff level analysis (MultiCoLA) was used to define abundant and rare taxa.^63^ According to the method procedure, the raw ASVs table was first sorted in descending order according to the number of sequences per ASV. Subsequently, low-abundance ASVs were truncated at successive cut-off levels (0, 1, 5–95, and 99%) of the total sum of ASV sequences. Then, the pairwise distance matrices were calculated from both the original and truncated data using the Bray-Curtis dissimilarity index, and these matrices were compared using the non-parametric Spearman’s rho correlation coefficient to assess their similarity. In addition, the Procrustes analysis was also applied to compare the non-metric multidimensional scaling (NMDS) ordination results from the original distance matrix with those from the truncated distance matrices. In this study, Procrustes' correlation value was used to assess the similarities of each truncated dataset between the original data at the phylum, class, order, family, genus, and ASV levels, because it can quantify the agreement between the main biological variation of extracted variation from the original versus truncated datasets. After analysis and defining 5% of the total number of reads as the rare threshold, the COtables R script was used to obtain the abundant and the rare ASVs table.

#### Measuring geographic distance between individuals

At each individual sampling point, spatial geographical coordinates and elevations were recorded by a handheld GPS (GPSmap 621sc, Garmin, Olathe, KS, USA). The pairwise geographic distance between each individual was calculated using the function distm in the geosphere (version 1.5–14) package,^64^ according to the GPS coordinates.

#### *α*-Diversity analysis

*α*-diversity indices, including observed ASVs (*S*_*obs*_), Shannon-Wiener index (Shannon), and Faith’s Phylogenetic Diversity (PD), were used to evaluate the microbial community richness, entropy, and phylogenetic diversity. All *α*-diversity indices were calculated using the R packages vegan^65^ and picante.^67^ The Kruskal-Wallis Rank-Sum Test was used to determine any significant difference by the host or population membership. Pairwise Wilcoxon Rank Sum Tests were used to determine any significant difference between each population, performed using the R package stats.^66^ Results were visualized using the R package ggplot2.^68^ Unless stated otherwise, the Benjamini-Hochberg method^78^ was used for multiple testing corrections, and *adjusted* p *values* ≤ 0.1 was considered significant.

Fisher’s exact test was performed with the R package stats and used to investigate the relationship between sympatric (or not) and significant differences between paired populations (or not).

A generalized linear mixed model (GLMM) was used to investigate the relationship between the response variable, alpha diversity of gut microbiota, and the explanatory variables, species and sympatry (whether the samples were collected from the same region). The sampling location was included as a random effect. The GLMM was fitted using the R package lme4.^69^

#### Distance-based analysis

Generalized UniFrac distances (GUniFrac) was used to perform all distance-based analyses in our study. The respective GUniFrac distances were calculated using the R package GUniFrac following the authors’ suggestion to set *d* = 0.5.^77^

Principal coordinate analysis (PCoA) based on GUniFrac distances was performed using the R package vegan, and the results were visualized using the R package ggplot2. Permutational multivariate analysis of variance (PERMANOVA) between Tibetan gazelle and Przewalski’s gazelle, and across populations, as well as pairwise PERMANOVAs test in any two groups of samples were performed with 9,999 permutations to test for any significant differences, all performed with the package vegan and visualized using the package ggplot2.

#### Mantel test

Due to the strong correlation among the spatial factors (longitude, latitude, and altitude), we first scaled each spatial factor, then performed principal components analysis (PCA) and extracted the PC1 score as the representative of the spatial factors for Mantel test, partial Mantel test, and multiple regression on matrices analysis (MRM).

The significance of the relationship between gut microbiota dissimilarity (GUniFrac distance) and PSM, spatial factors (PC1) in each host for all, abundant, and rare ASVs was assessed using the Mantel test with 9,999 permutations for each subset, which was implemented in the R package ecodist.^70^

#### Partial Mantel test

To disentangle separate influences of PSM, spatial factors (PC1), and geological distance on the gut microbiota composition in Tibetan gazelle and Przewalski’s gazelle at all ASVs, abundant ASVs, and rare ASVs, we performed partial Mantel tests using the R package ecodist.^70^ Specifically, the correlations (Spearman’s correlation) between bacterial GUniFrac distance (community dissimilarity) and geographical distance while controlling for spatial factors (PC1) distance and PSM was examined. Both the correlations between bacterial GUniFrac distance and spatial factors (PC1) while controlling for geographical distance and PSM, and between bacterial GUniFrac distance and PSM while controlling for geographic distance and spatial factors (PC1) distance in each subset were examined. All tests were performed with 9,999 permutations.

#### Multiple regression on matrices analysis

To further determine the relative importance of spatial factors (PC1) and PSM in structuring Przewalski’s gazelle bacterial communities, we conducted multiple regression on matrices (MRM) approach using package ecodist^70^ with 9,999 permutations.

#### iCAMP to analyze community assembly processes

The Infer Community Assembly Mechanisms by Phylogenetic-bin-based null model (iCAMP) was used to investigate the assembly mechanisms of gut microbiota in Tibetan gazelle and Przewalski’s gazelle. The iCAMP framework can identify both deterministic (homogeneous selection and heterogeneous selection) and stochastic processes (drift, dispersal limitation, and homogeneous dispersal) that contribute to the assembly of different phylogenetically related microbial groups (called ‘bins’).^71^ The most important parameter to perform iCAMP is to choose the proper minimal number of taxa in a bin (BinSize). Firstly, the stochasticity level was estimated using phylogenetic normalized stochasticity ratio (pNST), and then different BinSize (from 12 to 96) was tested, and BinSize = 12 for the final analysis was chosen because it had an estimated stochasticity similar to the pNST result. In addition, the changes in HoS, DL, and DR induced by host identity were investigated, and defined a change as a positive value if the relative contribution of HoS, DL, or DR was higher in Przewalski’s gazelle than in Tibetan gazelle.

#### Functional prediction and enrichment analysis

Functional prediction of the gut microbiota for Kyoto Encyclopedia of Genes and Genomes (KEGG)^52^ pathways was performed using the Phylogenetic Investigation of Communities by Reconstruction of Unobserved States (PICRUSt2) software,^72^ following the GitHub Wiki manual. This was based on the rarefied ASVs table and corresponding representative sequences. The ASVs that contributed to other processes (DR, DL, HD, and HeS) in Tibetan gazelle but the HoS process in Przewalski’s gazelle were extracted from iCAMP results and used for KEGG pathway enrichment analysis. The KEGG pathway enrichment analysis was performed by TBtools.^73^

Unless stated otherwise, all analyses were performed in R (version 4.1.3)^66^ and Rstudio (version 2021.09.0).^74^

## Supplemental information

Document S1. Figures S1‒S5 and Tables S1, S2, and S5‒S9

Table S3. Taxonomic classification of core ASVs in Tibetan gazelle and Przewalski’s gazelle, related to Figure 2

Table S4. Abundant and rare taxonomic composition of gut microbiota at phylum and family levels, related to Figure 2

Table S10. The characteristics of each bin, related to Figure 6

Table S11. Bin composition, related to Figure 6

Table S12. Dominant process of each bin in Tibetan gazelles and Przewalski’s Gazelles, related to Figure 6
