# Supplementary material for: Gut microbiota non-convergence and adaptations in sympatric Tibetan and Przewalski’s gazelles
Source: iScience. 2024 Feb 6;27(3):109117. doi: 10.1016/j.isci.2024.109117 (PMC10879710; doi:10.1016/j.isci.2024.109117)
Supplement: Document S1. Figures S1‒S5 and Tables S1, S2, and S5‒S9 [file mmc1.pdf]

iScience, Volume *27*

Supplemental information

Gut microbiota non-convergence and adaptations in sympatric Tibetan and Przewalski's gazelles

Pengfei Song, Feng Jiang, Daoxin Liu, Zhenyuan Cai, Hongmei Gao, Haifeng Gu, Jingjie Zhang, Bin Li, Bo Xu, and Tongzuo Zhang

Supplemental information

Figure S1. The upstream bioinformatics pipeline, related to the STAR Methods.

Figure S2. The rarefaction curves, related to the STAR Methods.

Figure S3. Identification of rare taxa threshold, related to Figure 1 and the STAR Methods.

Figure S4. Shared and unique taxa of the 6 populations, related to Figure 2.

Figure S5. Gut microbiota of sympatric gazelles did not converge, related to Figure 4.

Table S1. The detailed sample information, related to Figure 1.

Table S2. The detailed sample set, related to Figure 1.

Table S5. Kruskal-Wallis rank sum test across 2 species, related to Figure 3.

Table S6. Fisher exact test of independence (α – diversity), related to Figure 3.

Table S7. Generalized linear mixed model (GLMM) to test if host or sympatry predicted α – diversity, related to Figure 3.

Table S8. Fisher exact test of independence (β – diversity), related to Figure 4.

Table S9. Pairwise comparisons of soil-associated microbes across 6 populations using Wilcoxon rank sum test, related to Figure 4 and the STAR Methods.

Supplemental Material

Figure S1. The upstream bioinformatics pipeline, related to the STAR Methods

Solid blue boxes are conceptual steps and specific computational steps, green boxes indicate additional steps that conceptual steps are needed, and orange boxes indicate each step's main result. Bioinformatics pipelines were mainly performed with QIIME2-2022.2 framework.[S1] Raw sequence data were trimming adaptor and primer sequences using q2-cutadapt.[S2] DADA2 was adopted (via q2-dada2) to denoising sequences,[S3] optimized truncation length parameters were accessed by Figaro.[S4] The reference sequence annotation and curation pipeline (RESCRIPt)[S5] was used to prepare a QIIME2-compatible SILVA SSU 16S rRNA reference database, based on the SILVA curated NR99 (version 138.1) database.[S6] The V3 – V4 region of the 16S RNA gene was extracted and used to train an amplicon-specific naive-Bayes classifier to perform taxonomy classification via the q2-feature-classifier plugin. Taxonomy classification was done via Q2-feature-classifier classify-sklearn with a 0.8 confidence threshold.[S7] Taxonomy-based filtering was applied to remove all features that contain either mitochondria, chloroplast, or archaea, ASVs that are present in only 10% of total samples as well as the relative abundance less than millionth also be filtered. The filtered ASVs table was then normalized by rarefied to a minimum sequencing depth of all samples (depth = 26,685) to remove sample heterogeneity and used for all further analyses except FEAST analysis, which required the raw ASVs table. Finally, we used align-to-tree-mafft-fasttree pipeline from the q2-phylogeny plugin to generate a rooted phylogenetic tree.[S8,S9]

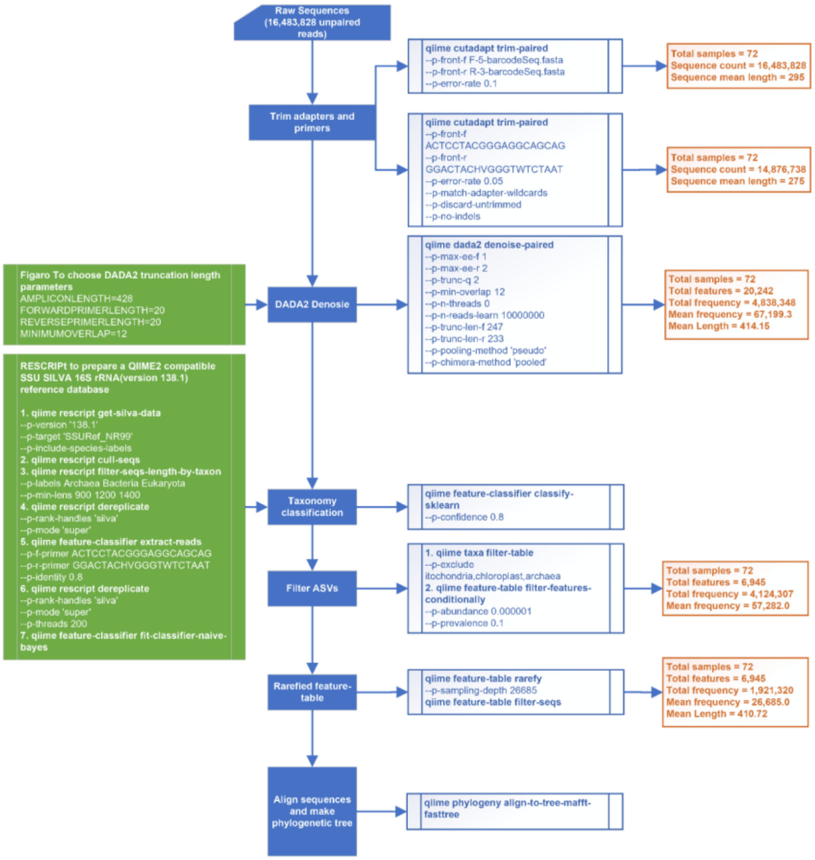

Figure S2. The rarefaction curves, related to the STAR Methods

The rarefaction curves of observed ASVs (a) and Shannon-Wiener index (b). The number of observed ASVs gradually plateaued as the sequencing depth increased, demonstrates that each sample had sufficient ASVs to reflect the maximum level of bacterial diversity.

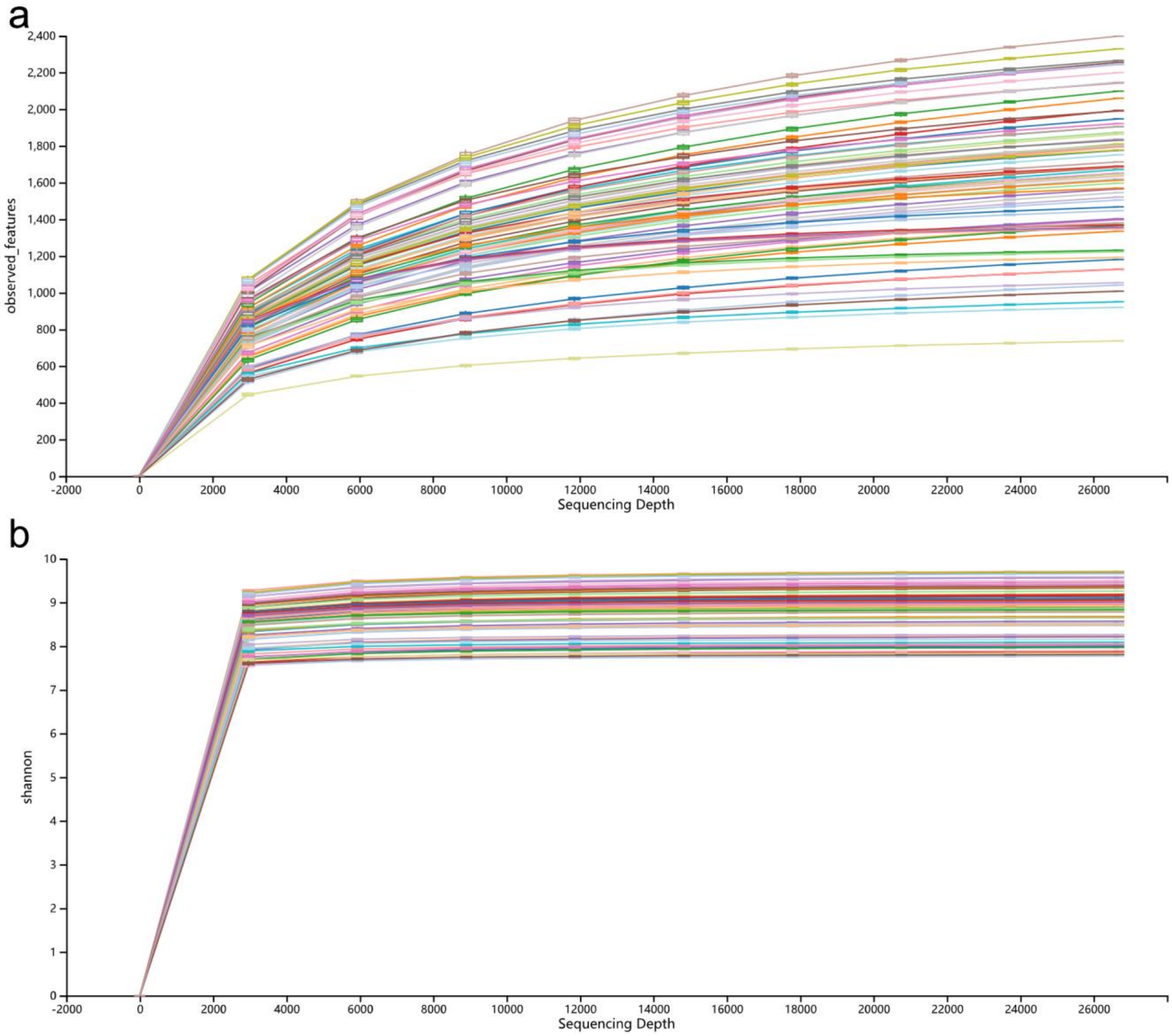

Figure S3. Identification of rare taxa threshold, related to Figure 1 and STAR Methods

The MultiCoLA analysis[S10] results. The removal of 5% of the sequences from the original data set resulted in a variation of the Procrustes correlation between the truncated data sets and original data set in Tibetan gazelle and Przewalski's gazelle were also decreasing, especially at phylum and order level.

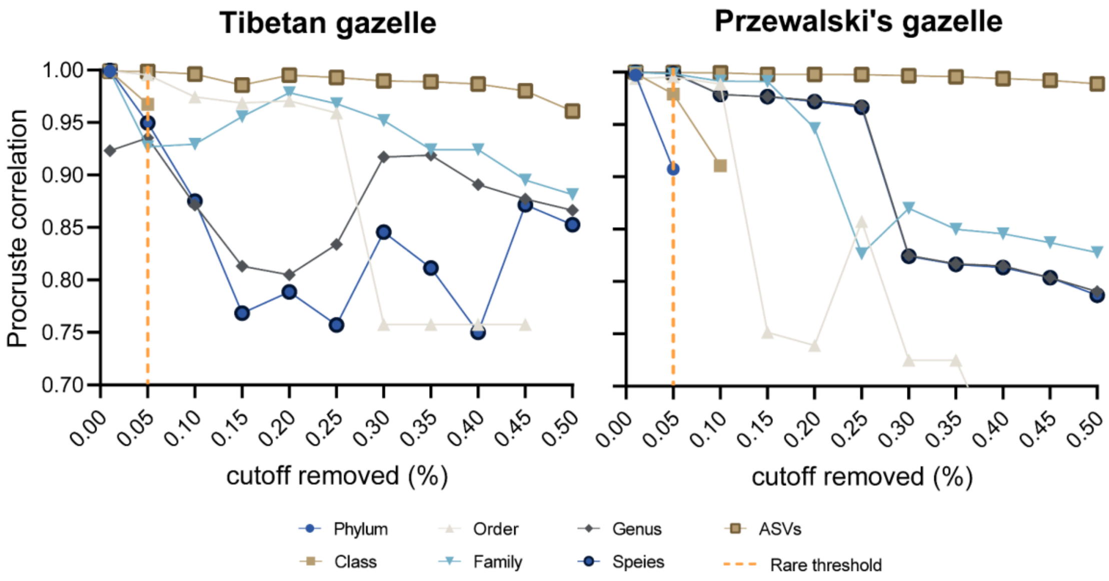

Figure S4. Shared and unique taxa of the 6 populations, related to Figure 2

Shared and unique (a) abundant phyla, (b) rare phyla, (c) abundant families, and (d) rare families among 6 populations of 2 gazelles.

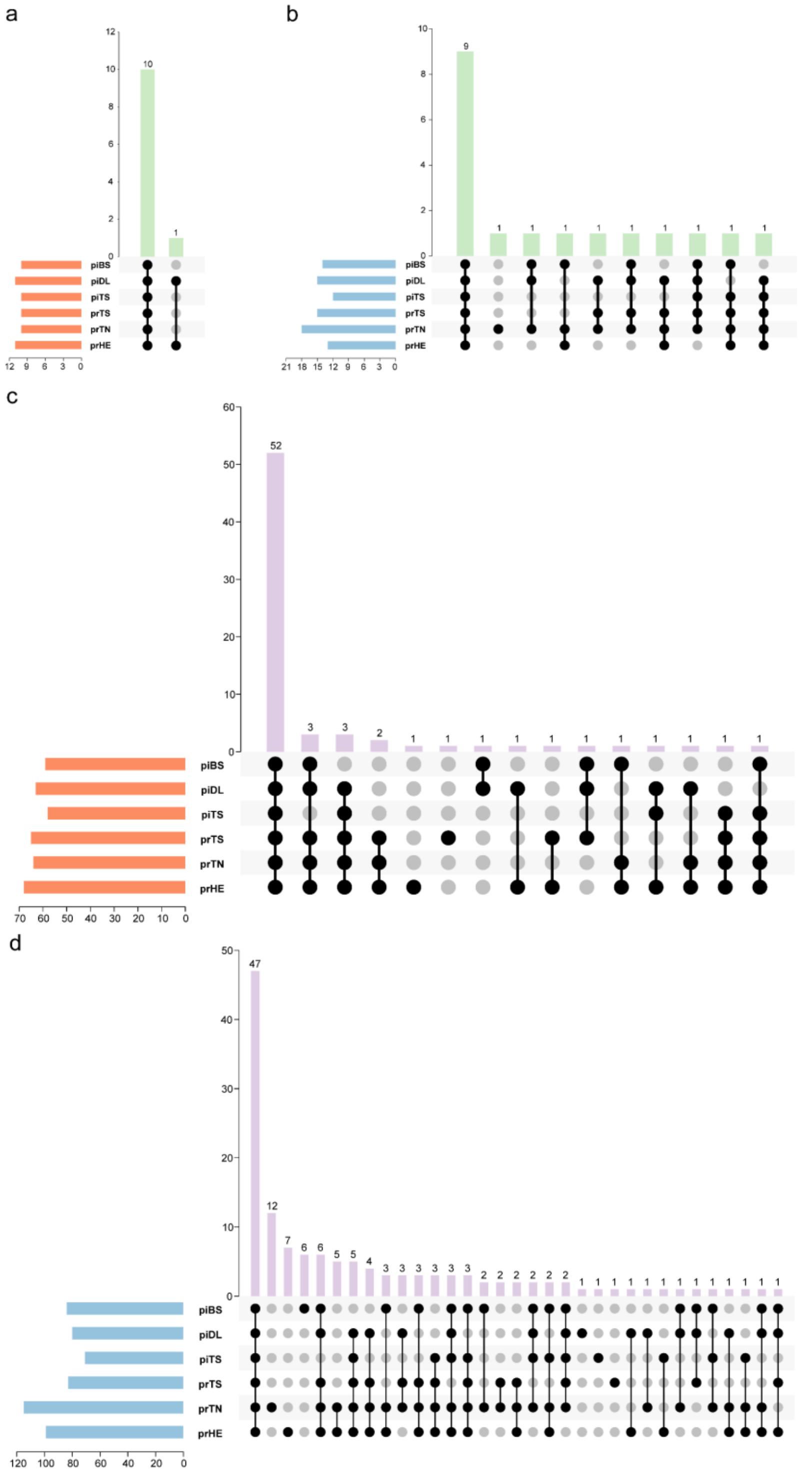

Figure S5. Gut microbiota of sympatric gazelles did not converge, related to Figure 4

Individuals' gut microbiota from different pairs of host populations is shown as the average pairwise[S11] dissimilarities (d = 0.5) in each bar. The average pairwise Generalized UniFrac dissimilarities of (a) abundant ASVs, and (b) rare ASVs, data are represented as mean ± SD. The post hoc test used Fisher's least significant difference (LSD) test with Bonferroni correction (*adjusted p-values* < 0.05).

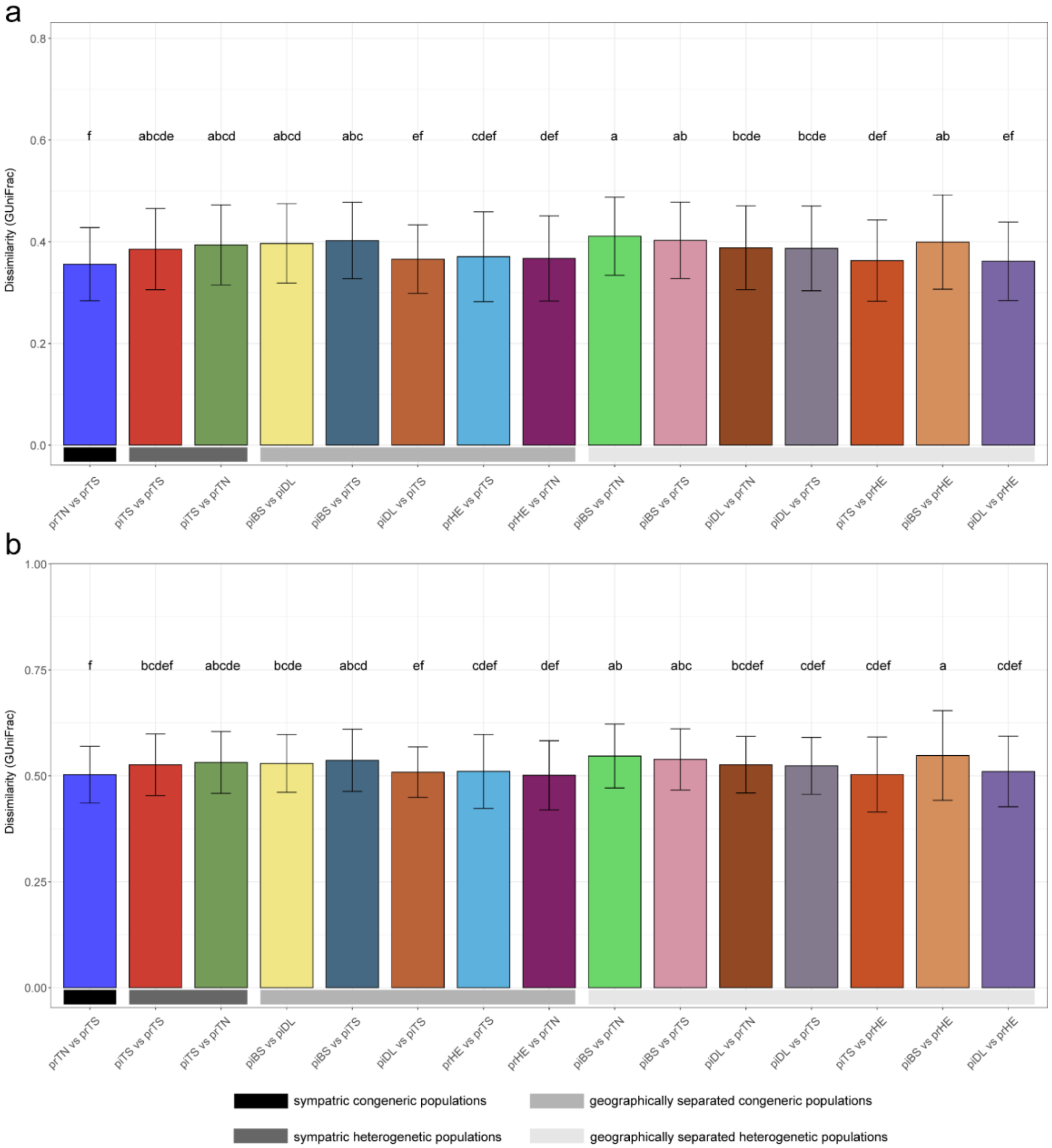

Table S1. The detailed sample information, related to Figure 1

Populations	Sample.id	Host	Collection site	Latitude	Longitude	Altitude	Collection date	Sympatric
piBS (n=10)	piBS_01	*Procapra picticaudata*	BS	37.61	97.02	3753.67	2021/12/4	F
	piBS_02	*Procapra picticaudata*	BS	37.61	97.02	3754.06	2021/12/4	
	piBS_03	*Procapra picticaudata*	BS	37.61	97.02	3754.07	2021/12/4	
	piBS_04	*Procapra picticaudata*	BS	37.61	97.02	3756.53	2021/12/4	
	piBS_05	*Procapra picticaudata*	BS	37.61	97.02	3756.55	2021/12/4	
	piBS_06	*Procapra picticaudata*	BS	37.61	97.02	3763.79	2021/12/4	
	piBS_07	*Procapra picticaudata*	BS	37.61	97.02	3771.15	2021/12/4	
	piBS_08	*Procapra picticaudata*	BS	37.61	97.02	3771.02	2021/12/4	
	piBS_09	*Procapra picticaudata*	BS	37.61	97.02	3768.10	2021/12/4	
	piBS_10	*Procapra picticaudata*	BS	37.61	97.02	3765.55	2021/12/4	
sBS (n=3)	sBS_01	soil	BS	37.61	97.02	3756.55	2021/12/4	\
	sBS_02	soil	BS	37.61	97.02	3756.55	2021/12/4	
	sBS_03	soil	BS	37.61	97.02	3756.55	2021/12/4	
piDL (n=10)	piDL_01	*Procapra picticaudata*	DL	37.63	96.70	4004.41	2021/12/4	F
	piDL_02	*Procapra picticaudata*	DL	37.63	96.70	4004.54	2021/12/4	
	piDL_03	*Procapra picticaudata*	DL	37.63	96.70	4003.31	2021/12/4	
	piDL_04	*Procapra picticaudata*	DL	37.63	96.70	4007.27	2021/12/4	
	piDL_05	*Procapra picticaudata*	DL	37.63	96.70	4007.77	2021/12/4	
	piDL_06	*Procapra picticaudata*	DL	37.63	96.70	4007.76	2021/12/4	
	piDL_07	*Procapra picticaudata*	DL	37.63	96.70	4008.68	2021/12/4	
	piDL_08	*Procapra picticaudata*	DL	37.63	96.70	4008.67	2021/12/4	
	piDL_09	*Procapra picticaudata*	DL	37.63	96.70	4008.66	2021/12/4	
	piDL_10	*Procapra picticaudata*	DL	37.63	96.70	4008.66	2021/12/4	
sDL (n=3)	sDL_01	soil	DL	37.63	96.70	4007.76	2021/12/4	\
	sDL_02	soil	DL	37.63	96.70	4007.76	2021/12/4	
	sDL_03	soil	DL	37.63	96.70	4007.76	2021/12/4	
piTS (n=7)	piTS_01	*Procapra picticaudata*	TS	37.48	98.54	3638.95	2021/12/11	T
	piTS_02	*Procapra picticaudata*	TS	37.48	98.54	3626.52	2021/12/11	
	piTS_03	*Procapra picticaudata*	TS	37.48	98.54	3626.50	2021/12/11	
	piTS_04	*Procapra picticaudata*	TS	37.48	98.54	3629.08	2021/12/11	
	piTS_05	*Procapra picticaudata*	TS	37.48	98.54	3629.08	2021/12/11	
	piTS_06	*Procapra picticaudata*	TS	37.48	98.54	3629.08	2021/12/11	
	piTS_07	*Procapra picticaudata*	TS	37.48	98.54	3629.08	2021/12/11	
sTS (n=3)	sTS_01	soil	TS	37.48	98.54	3629.08	2021/12/11	\
	sTS_02	soil	TS	37.48	98.54	3629.08	2021/12/11	

	sTS_03	soil	TS	37.48	98.54	3629.08	2021/12/11	
prHE (n=10)	prHE_01	*Procapra przewalskii*	HE	37.22	100.45	3267.70	2021/12/11	F
	prHE_02	*Procapra przewalskii*	HE	37.22	100.45	3267.65	2021/12/11	
	prHE_03	*Procapra przewalskii*	HE	37.22	100.45	3267.66	2021/12/11	
	prHE_04	*Procapra przewalskii*	HE	37.22	100.45	3267.64	2021/12/11	
	prHE_05	*Procapra przewalskii*	HE	37.22	100.45	3267.64	2021/12/11	
	prHE_06	*Procapra przewalskii*	HE	37.22	100.45	3267.64	2021/12/11	
	prHE_07	*Procapra przewalskii*	HE	37.22	100.45	3267.64	2021/12/11	
	prHE_08	*Procapra przewalskii*	HE	37.22	100.45	3267.65	2021/12/11	
	prHE_09	*Procapra przewalskii*	HE	37.22	100.45	3267.64	2021/12/11	
	prHE_10	*Procapra przewalskii*	HE	37.22	100.45	3267.65	2021/12/11	
sHE (n=3)	sHE_01	soil	HE	37.22	100.45	3267.64	2021/12/11	\
	sHE_02	soil	HE	37.22	100.45	3267.64	2021/12/11	
	sHE_03	soil	HE	37.22	100.45	3267.64	2021/12/11	
PrTN (n=10)	prTN_01	*Procapra przewalskii*	TN	37.49	98.53	3665.38	2021/12/11	T
	prTN_02	*Procapra przewalskii*	TN	37.49	98.53	3664.28	2021/12/11	
	prTN_03	*Procapra przewalskii*	TN	37.49	98.53	3664.29	2021/12/11	
	prTN_04	*Procapra przewalskii*	TN	37.49	98.53	3664.29	2021/12/11	
	prTN_05	*Procapra przewalskii*	TN	37.49	98.53	3664.29	2021/12/11	
	prTN_06	*Procapra przewalskii*	TN	37.49	98.53	3664.29	2021/12/11	
	prTN_07	*Procapra przewalskii*	TN	37.49	98.53	3664.29	2021/12/11	
	prTN_08	*Procapra przewalskii*	TN	37.49	98.53	3664.29	2021/12/11	
	prTN_09	*Procapra przewalskii*	TN	37.49	98.53	3664.29	2021/12/11	
	prTN_10	*Procapra przewalskii*	TN	37.49	98.53	3664.30	2021/12/11	
sTN (n=3)	sTN_01	soil	TN	37.49	98.53	3664.29	2021/12/11	\
	sTN_02	soil	TN	37.49	98.53	3664.29	2021/12/11	
	sTN_03	soil	TN	37.49	98.53	3664.29	2021/12/11	
prTS (n=10)	prTS_01	*Procapra przewalskii*	TS	37.47	98.53	3676.21	2021/12/11	T
	prTS_02	*Procapra przewalskii*	TS	37.47	98.53	3676.22	2021/12/11	
	prTS_03	*Procapra przewalskii*	TS	37.47	98.53	3664.64	2021/12/11	
	prTS_04	*Procapra przewalskii*	TS	37.47	98.53	3664.64	2021/12/11	
	prTS_05	*Procapra przewalskii*	TS	37.48	98.53	3646.81	2021/12/11	
	prTS_06	*Procapra przewalskii*	TS	37.48	98.53	3646.82	2021/12/11	
	prTS_07	*Procapra przewalskii*	TS	37.48	98.53	3646.82	2021/12/11	
	prTS_08	*Procapra przewalskii*	TS	37.48	98.53	3658.01	2021/12/11	
	prTS_09	*Procapra przewalskii*	TS	37.48	98.53	3664.40	2021/12/11	
	prTS_10	*Procapra przewalskii*	TS	37.47	98.53	3670.99	2021/12/11	

Table S2. The detailed sample set, related to Figure 1

Sympatric		Allopatric	
**Congeneric**	**Heterogenetic**	**Congeneric**	**Heterogenetic**
prTN Vs. prTS	piTS Vs. prTS	piBS Vs. piDL	piBS Vs. prTN
	piTS Vs. prTN	piBS Vs. piTS	piBS Vs. prTS
		piDL Vs. piTS	piDL Vs. prTN
		prHE Vs. prTS	piDL Vs. prTS
		prHE Vs. prTN	piTS Vs. prHE
			piBS Vs. prHE
			piDL Vs. prHE

Table S5. Kruskal-Wallis rank sum test across 2 species, related to Figure 3

	All ASVs			Abundant ASVs			Rare ASVs		
	H	df	*p*	*H*	df	*p*	*H*	df	*p*
S_{obs}	34.60	1	0.00000000	34.59	1	0.00000000	21.04	1	0.00000449
Shannon	24.23	1	0.00000085	24.39	1	0.00000079	25.99	1	0.00000034
PD	28.15	1	0.00000011	28.67	1	0.00000009	23.45	1	0.00000128

Table S6. Fisher exact test of independence (α – diversity), related to Figure 3

		Sympatric distribution pairwise comparisons	Non-sympatric distribution pairwise comparisons	Fisher's exact P-value
S_{obs} (all ASVs)	Significant pairwise comparisons	2	11	0.3714
	Non-significant pairwise comparisons	1	1	
S_{obs} (abundant ASVs)	Significant pairwise comparisons	2	11	0.3714
	Non-significant pairwise comparisons	1	1	
S_{obs} (rare ASVs)	Significant pairwise comparisons	2	9	1
	Non-significant pairwise comparisons	1	3	
shannon (all ASVs)	Significant pairwise comparisons	1	10	0.1538
	Non-significant pairwise comparisons	2	2	
shannon (abundant ASVs)	Significant pairwise comparisons	1	10	0.1538
	Non-significant pairwise comparisons	2	2	
shannon (rare ASVs)	Significant pairwise comparisons	1	11	0.08132
	Non-significant pairwise comparisons	2	1	
faithPD (all ASVs)	Significant pairwise comparisons	2	10	0.5165
	Non-significant pairwise comparisons	1	2	
faithPD (abundant ASVs)	Significant pairwise comparisons	2	10	0.5165
	Non-significant pairwise comparisons	1	2	
faithPD (rare ASVs)	Significant pairwise comparisons	2	11	0.3714
	Non-significant pairwise comparisons	1	1	

Table S7. Generalized linear mixed model (GLMM) to test if host or sympatry predicted α – diversity related to Figure 3

		BIC	R^2 (conditional)	R^2 (marginal)	Sigma		Wald Chi-Square (t-value)	p
S_{obs}	All ASVs	727.59	0.84	0.41	141.89	host	2.35	0.02
						sympatry	0.42	0.67
	Abundant ASVs	686.58	0.83	0.43	97.25	host	2.33	0.02
						sympatry	0.12	0.91
	Rare ASVs	633.51	0.78	0.35	59.82	host	2.18	0.03
						sympatry	0.93	0.36
Shannon	All ASVs	67.45	0.71	0.27	0.32	host	1.73	0.09
						sympatry	0.05	0.96
	Abundant ASVs	61.87	0.69	0.26	0.30	host	1.69	0.10
						sympatry	-0.08	0.94
	Rare ASVs	35.44	0.71	0.36	0.24	host	2.38	0.02
						sympatry	0.66	0.51
PD	All ASVs	329.32	0.82	0.33	3.55	host	1.99	0.05
						sympatry	0.44	0.66
	Abundant ASVs	277.09	0.85	0.29	2.17	host	1.71	0.09
						sympatry	0.21	0.83
	Rare ASVs	317.31	0.80	0.33	3.19	host	2.04	0.05
						sympatry	0.66	0.51

Table S8. Fisher exact test of independence (β – diversity), related to Figure 4

		Sympatric distribution pairwise comparisons	Non-sympatric distribution pairwise comparisons	Fisher's exact P-value
Gunifrac distance (all ASVs)	Significant pairwise comparisons	3	12	1
	Non-significant pairwise comparisons	0	0	
Gunifrac distance (abundant ASVs)	Significant pairwise comparisons	3	12	1
	Non-significant pairwise comparisons	0	0	
Gunifrac distance (rare ASVs)	Significant pairwise comparisons	3	12	1
	Non-significant pairwise comparisons	0	0	

Table S9. Pairwise comparisons of soil-associated microbes across 6 populations using Wilcoxon rank sum test, related to Figure 4 and the STAR Methods

	piBS	piDL	piTS	prHE	prTN
piDL	0.060				
piTS	0.004	0.041			
prHE	0.132	0.003	0.003		
prTN	0.001	0.032	0.887	0.000	
prTS	0.017	0.002	0.002	0.017	0.000

*P values were adjusted using Benjamini-Hochberg method. Pairwise difference consider significant at adjusted $p \leq 0.1$.

Supplemental references

[S1] Bolyen, E., Rideout, J.R., Dillon, M.R., Bokulich, N.A., Abnet, C.C., Al-Ghalith, G.A., Alexander, H., Alm, E.J., Arumugam, M., Asnicar, F., et al. (2019). Reproducible, interactive, scalable and extensible microbiome data science using QIIME 2. Nat Biotechnol 37, 852–857. 10.1038/s41587-019-0209-9.

[S2] Martin, M. (2011). Cutadapt removes adapter sequences from high-throughput sequencing reads. EMBnet.journal 17, 10–12. 10.14806/ej.17.1.200.

[S3] Callahan, B.J., McMurdie, P.J., Rosen, M.J., Han, A.W., Johnson, A.J.A., and Holmes, S.P. (2016). DADA2: High-resolution sample inference from Illumina amplicon data. Nat Methods 13, 581–583. 10.1038/nmeth.3869.

[S4] Weinstein, M.M., Prem, A., Jin, M., Tang, S., and Bhasin, J.M. (2019). FIGARO: An efficient and objective tool for optimizing microbiome rRNA gene trimming parameters. Preprint at bioRxiv, 10.1101/610394 10.1101/610394.

[S5] Robeson, M.S., O'Rourke, D.R., Kaehler, B.D., Ziemski, M., Dillon, M.R., Foster, J.T., and Bokulich, N.A. (2021). RESCRIPt: Reproducible sequence taxonomy reference database management. PLoS computational biology 17, e1009581. 10.1371/journal.pcbi.1009581.

[S6] Quast, C., Pruesse, E., Yilmaz, P., Gerken, J., Schweer, T., Yarza, P., Peplies, J., and Glöckner, F.O. (2013). The SILVA ribosomal RNA gene database project: improved data processing and web-based tools. Nucleic Acids Res 41, D590–D596. 10.1093/nar/gks1219.

[S7] Bokulich, N.A., Kaehler, B.D., Rideout, J.R., Dillon, M., Bolyen, E., Knight, R., Huttley, G.A., and Gregory Caporaso, J. (2018). Optimizing taxonomic classification of marker-gene amplicon sequences with QIIME 2's q2-feature-classifier plugin. Microbiome 6, 90. 10.1186/s40168-018-0470-z.

[S8] Katoh, K., and Standley, D.M. (2013). MAFFT Multiple Sequence Alignment Software Version 7: Improvements in Performance and Usability. Molecular Biology and Evolution 30, 772–780. 10.1093/molbev/mst010.

[S9] Price, M.N., Dehal, P.S., and Arkin, A.P. (2010). FastTree 2-Approximately Maximum-Likelihood Trees for Large Alignments. PLoS One 5, e9490. 10.1371/journal.pone.0009490.

[S10] Gobet, A., Quince, C., and Ramette, A. (2010). Multivariate Cutoff Level Analysis (MultiCoLA) of large community data sets. Nucleic Acids Res 38, e155. 10.1093/nar/gkq545.

[S11] Chen, J., Bittinger, K., Charlson, E.S., Hoffmann, C., Lewis, J., Wu, G.D., Collman, R.G., Bushman, F.D., and Li, H. (2012). Associating microbiome composition with environmental covariates using generalized UniFrac distances. Bioinformatics 28, 2106–2113. 10.1093/bioinformatics/bts342.
